# Amphiregulin and Epiregulin Confer Radioresistance in Esophageal Squamous Cell Carcinoma Through Oxidative Phosphorylation

**DOI:** 10.1002/advs.202507524

**Published:** 2025-11-09

**Authors:** Zhang Lin, Meilian Yao, Xin Xu, Dong Zhang, Lei Xu, Ling Rong, Xiaohang Wang, Yi Duan, Chengkun Chen, Jun Gu, Yao Zhang, Qiang Liu, Qing Ye, Gaoping Cui, Yujun Hao, Xiumei Ma

**Affiliations:** ^1^ State Key Laboratory of Systems Medicine for Cancer Shanghai Cancer Institute Renji Hospital School of Medicine Shanghai Jiao Tong University Shanghai 200032 China; ^2^ Department of Radiation Oncology Renji Hospital School of Medicine Shanghai Jiao Tong University Shanghai 200127 China; ^3^ Department of Gastroenterology Renji Hospital School of Medicine Shanghai Jiao Tong University Shanghai 200127 China; ^4^ Department of Pathology Renji Hospital School of Medicine Shanghai Jiao Tong University Shanghai 200127 China; ^5^ Department of Thoracic Surgery Renji Hospital School of Medicine Shanghai Jiao Tong University Shanghai 200127 China

**Keywords:** esophageal squamous cell carcinoma, neoadjuvant chemoradiotherapy, OXPHOS, CEBPB, AREG, EREG

## Abstract

Neoadjuvant chemoradiotherapy (nCRT) improves outcomes for locally advanced esophageal squamous cell carcinoma (ESCC) but exhibits variable efficacy due to heterogeneous radiosensitivity. In this study, metabolic profiling of 59 ESCC patients revealed significant alterations in tricarboxylic acid cycle (TCA) cycle intermediates in pathological complete responders (pCR) vs. non‐responders (non‐pCR) ESCC patients after nCRT. Functional experiments demonstrated that radioresistant ESCC cells (KYSE410R) exhibited elevated OXPHOS activity, which is reversed by targeting TCA cycle enzymes (CPI‐613 (Devimistat), fumarate hydratase‐IN‐1 (FH‐IN‐1), etc.) or Oxidative phosphorylation (OXPHOS) inhibitors (IACS‐010759, Rotenone, etc.). Irradiation‐induced CCAAT Enhancer Binding Protein Beta (CEBPB) upregulated AREG/EREG expression, activating the ERBB/mTOR pathway to promote OXPHOS flux. Knockdown of CEBPB/AREG/EREG disrupted OXPHOS and sensitized ESCC cells to radiation. Clinically, high Amphiregulin/Epiregulin (AREG/EREG) levels correlated with nCRT resistance and poor prognosis. Collectively, the CEBPB/AREG/EREG axis drives radioresistance by reprogramming OXPHOS, suggesting inhibition of this pathway or OXPHOS itself as a promising strategy to enhance ESCC therapeutic responses.

## Graphical abstract

Oxidative phosphorylation (OXPHOS) activity serves as a critical metabolic determinant of radiotherapy outcomes in esophageal squamous cell carcinoma (ESCC). The CEBPB/AREG/EREG axis drives radioresistance in ESCC by reprogramming OXPHOS. AREG and EREG function as predictive biomarkers for nCRT response and prognosis of ESCC patients. Targeting ERBBs or OXPHOS enhances IR efficacy, providing novel treatment strategies for ESCC patients.

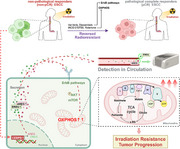

## 1 Introduction

Esophageal carcinoma (ESCA) ranks as the eleventh most prevalent malignancy worldwide and represents the seventh leading cause of cancer‐associated mortality,^[^ ^1^ ^]^ and esophageal squamous cell carcinoma (ESCC) accounts for 80% of all esophageal cancers in East Asian.^[^ ^2^, ^3^ ^]^ Neoadjuvant chemoradiotherapy (nCRT) followed by surgery is a standard approach for locally advanced ESCC. However, the efficacy of nCRT for ESCC varies greatly among individuals. Up to 30–50% of nCRT patients achieve pathological complete response (pCR) and long‐term survival,^[^ ^4^, ^5^ ^]^ while a considerable number of patients experience short‐term recurrence or even progression disease.^[^ ^6^ ^]^ Notably, emerging evidence suggests similar overall survival outcomes between patients achieving clinical complete response who decline surgery and those undergoing standard surgical resection after nCRT,^[^ ^7^, ^8^, ^9^, ^10^ ^]^ suggesting that extended nCRT can be a feasible approach for organ preservation of these patients. Given the critical role of irradiation (IR) in optimizing the efficacy of nCRT, there is growing emphasis on elucidating the molecular mechanisms and identifying predictive biomarkers of radiosensitivity in ESCC patients.

Metabolic reprogramming, a hallmark of cancer, enables ESCC cells to develop radioresistance through adaptive plasticity. The glycolysis pathway appears particularly crucial for IR response. IR‐induced upregulation of glycolytic enzymes (glucose transporter 1(GLUT1), hexokinase 2 (HK2) and pyruvate dehydrogenase kinase 1 (PDK1)) enhances ESCC glycolytic capacity and radioresistance of cancer cells.^[^ ^11^ ^]^ As a consequence, the accumulation of lactic acid, a major substrate of glycolysis, was correlated with radioresistance.^[^ ^12^ ^]^ High expression of tricarboxylic acid cycle (TCA cycle) enzyme IDH2 (isocitrate dehydrogenase 2) also confers resistance to IR for ESCA.^[^ ^13^ ^]^ Moreover, several metabolites have been identified as biomarkers to predict nCRT response. Xu et al. discovered that three lipids, including octanoylcarnitine, lysoPC (16:1), and decanoylcarnitine in plasma, can predict the nCRT response.^[^ ^14^ ^]^ Fujigaki et al. found that amino acids such as glycine, L‐serine, and L‐arginine can be applied to differentiate nCRT pCR from non‐pCR ESCC patients.^[^ ^15^ ^]^ Nevertheless, Zhang et al. showed that isocitric acid, linoleic acid, citric acid, L‐histidine, and 3′ 4‐dihydroxyhydrocinnamic acid are differentially expressed in nCRT pCR from non‐pCR ESCC patients.^[^ ^16^ ^]^ However, the pivotal metabolic mechanisms for ESCC radioresistance still remain inconclusive.

Radiation‐responsive metabolic pathways are frequently modulated by oncogenic signaling cascades. PI3K/AKT pathway regulates GLUT1 expression to confer the radioresistance of cancer cells, thereby targeting PI3K/AKT/mTOR effectively improves their sensitivity to irradiation.^[^ ^17^, ^18^, ^19^ ^]^ BRAF/ERK1/2 axis modulates lactate dehydrogenase A (LDHA)‐mediated lactate metabolism in prostate cancer radioresponse.^[^ ^20^ ^]^ Hypoxia modulates the expression of multiple glycolytic enzymes to affect the radiotherapy sensitivity of tumor cells.^[^ ^21^, ^22^, ^23^ ^]^ However, how metabolism is regulated by oncogenic signaling in ESCC regarding radioresistance remains poorly characterized.

In this study, we discovered that oxidative phosphorylation (OXPHOS) activity is critical for the radiosensitivity of ESCC. Amphiregulin (AREG) and Epiregulin (EREG), the ligands for ERBB receptors, regulate OXPHOS to confer radioresistance in ESCC. Targeting OXPHOS or ErbB pathways enhances the radiosensitivity of ESCC, and AREG and EREG serve as biomarkers to determine nCRT sensitivity for ESCC patients.

## 2 Results

### 2.1 TCA Cycle Intermediates are Disturbed in nCRT‐Responsive ESCC Patients

**Figure 1:**
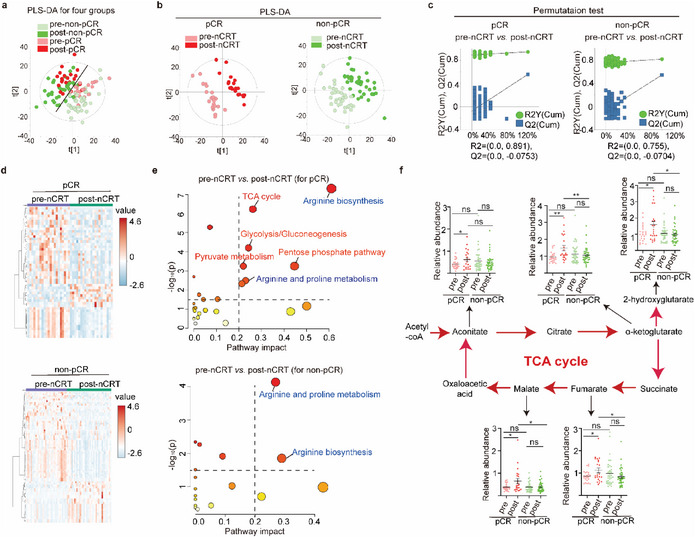
Alterations in TCA cycle intermediates characterize nCRT‐responsive ESCC patients. a–c) Metabolic profiles exhibit stronger nCRT‐induced perturbations in pCR compared to non‐pCR ESCC patients. a) PLS‐DA models comparing serum metabolic profiles across four experimental groups: pre‐pCR (serum from pCR patients before nCRT, *N* = 23), post‐pCR (serum from pCR patients after nCRT, *N* = 23), pre‐non‐pCR (serum from non‐pCR patients before nCRT, *N* = 36), and post‐non‐pCR (serum from non‐pCR patients after nCRT, *N* = 36). b) Stratified PLS‐DA models analyzing pre‐ vs. post‐nCRT metabolic profiles within pCR and non‐pCR cohorts. c) Permutation tests validate PLS‐DA models of (b). d) Heatmaps of differentially expressed metabolites (DEMs) between pre‐nCRT and post‐nCRT in pCR or non‐pCR ESCC patients, respectively. e) KEGG pathway enrichment analyses of DEMs between pre‐nCRT and post‐nCRT in pCR or non‐pCR ESCC patients, respectively. *Y* axis represents ‐lg (*p* value) of pathway analysis. *X* axis represents the impact factor (amount of DEMs enriched in the pathway/amount of all metabolites in the pathway). f) The abundance of TCA cycle intermediates was increased in the pCR group, but not in the non‐pCR group, after nCRT. Student's *t*‐test was used for statistical analyses. Data are presented as mean ± SEM. * *p* < 0.05, ** *p* < 0.01.

According to pathological results of surgical specimens, 59 ESCC patients were evaluated and divided into pCR or non‐pCR groups in response to nCRT (Table S1, Supporting Information). To identify the metabolic differences between nCRT pCR and nCRT non‐pCR ESCC patients, serum samples of a total 59 ESCC patients were collected before (pre‐) and after (post‐) nCRT treatment. Metabolome of serum samples of four groups (pre‐pCR, post‐pCR, pre‐non‐pCR, post‐non‐pCR) was performed with both untargeted ultra‐high‐performance liquid chromatography‐quadrupole time‐of‐flight mass spectrometry (UHPLC‐QTOF MS) and targeted ultra‐high‐performance liquid chromatography‐QTRAP mass spectrometry (UHPLC‐QTRAP MS) metabolomics, and a total ≈300 metabolites were analyzed. The metabolic profiles of four groups were not well‐separated in the unsupervised principal component analysis (PCA) score plots (Figure S1a, Supporting Information). The supervised partial least squares‐discriminant analysis (PLS‐DA) model showed that the pCR group and the non‐pCR group had no obvious differences either pre‐ or post‐nCRT (**Figure** **1**a), however, nCRT treatment tended to differentiate metabolic profiles of both the pCR group and the non‐pCR group (Figure 1a; Figure S1b, Supporting Information). Then metabolic differences of the four groups were further analyzed pairwise with the PLS‐DA model. Consistently, neither pre‐nCRT nor post‐nCRT metabolite profiles were not well‐separated between the pCR group and the non‐pCR group (Figure S1c,d, Supporting Information). However, metabolite profiles were well‐separated between pre‐nCRT and post‐nCRT for both the pCR group and the non‐pCR group (Figure 1b), and permutation tests showed that PLS‐DA models were well fitted (Figure 1c). Most importantly, pCR group showed greater separation than the non‐pCR group in terms of metabolic difference between pre‐nCRT and post‐nCRT (Figure 1b,c), suggesting that nCRT has tremendous influences on the metabolome of ESCC patients, especially for pCR patients.

To explore the metabolic mechanisms that influence the efficacy of nCRT, the differentially expressed metabolites (DEMs) of pre‐pCR vs. post‐pCR and pre‐non‐pCR vs. post‐non‐pCR were further analyzed, and metabolic pathways were enriched by KEGG (Figure 1d,e; Tables S2 and S3, Supporting Information). Amino acid pathways were disturbed by nCRT for both the pCR and non‐pCR group, such as arginine biosynthesis (Figure 1e; Tables S4 and S5, Supporting Information). Interestingly, the TCA cycle and related pathways, including glycolysis and pyruvate metabolism, were disturbed by nCRT only in the pCR group (Figure 1e; Tables S4 and S5, Supporting Information). The relative abundance of TCA cycle and related intermediates (aconitate, α‐ketoglutarate, malate, fumarate, and 2‐hydroxyglutarate) was increased in the pCR group after nCRT but was not altered in the non‐pCR group after nCRT (Figure 1f), suggesting that the TCA cycle is disturbed in ESCC patients who achieve pathological complete response by nCRT.

### 2.2 Inhibition of OXPHOS Enhances Radiosensitivity of ESCC Cells

**Figure 2:**
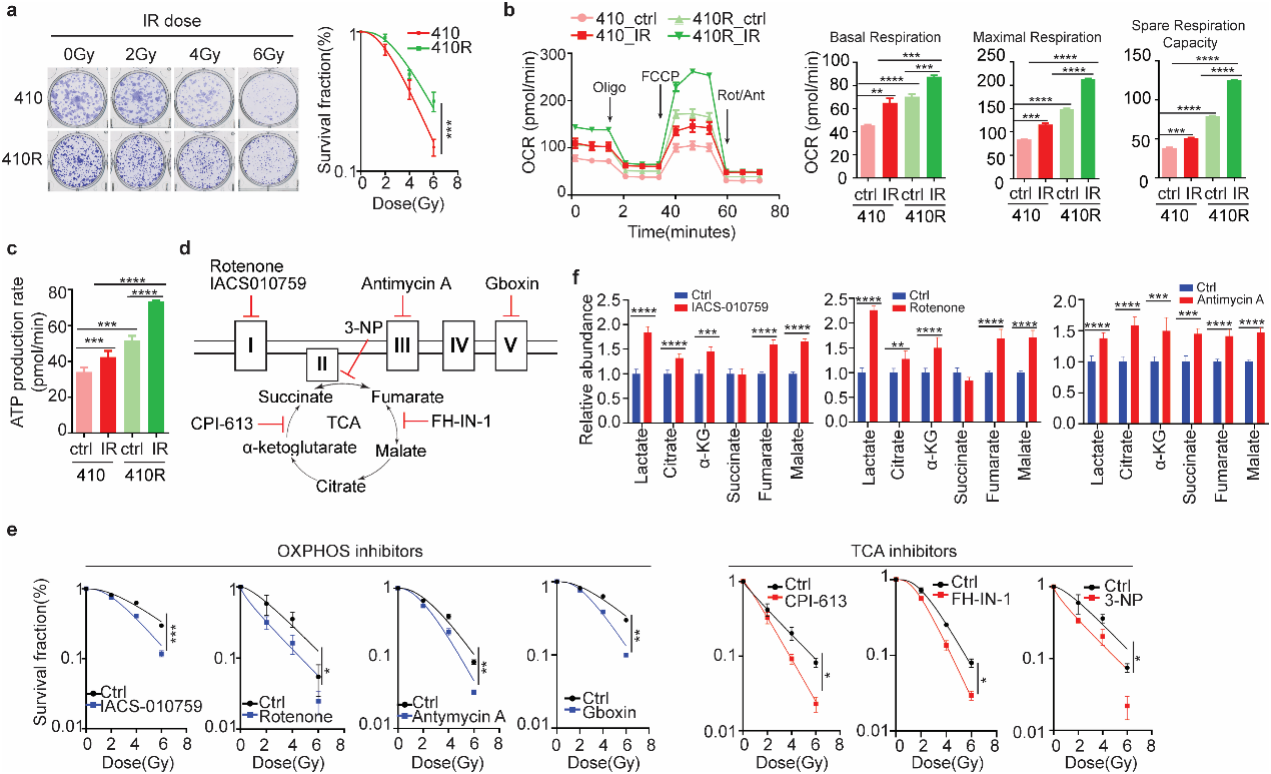
Inhibition of OXPHOS enhances the radiosensitivity of ESCC cells. a) Colony formation survival fractions of parental KYSE410 (410) and radioresistant KYSE410R (410R) cells post‐irradiation. b) Oxygen Consumption Rate (OCR) trace was determined using Seahorse XF96 analyzer. Oligo: oligomycin; FCCP: carbonyl cyanide‐4‐phenyl‐hydrazone; Rot/Ant: Rotenone/Antimycin A. c) ATP generation was analyzed by Seahorse analyzer. d Schematic of inhibitors targeting TCA cycle (CPI‐613, 3‐NP, FH‐IN‐1) and OXPHOS (IACS‐010759, Rotenone, Gboxin, Antimycin A). e) Radiosensitization effects of TCA cycle or OXPHOS inhibitors on KYSE30 cells were assessed by colony formation survival fraction. f) Relative abundance of TCA cycle intermediates in KYSE30 cells with or without OXPHOS inhibitors was analyzed by UHPLC‐QTRAP MS (*N* = 6). Two‐way ANOVA was used for statistical analyses of a, e. Student's *t*‐test was used for b, c, f. Data are presented as mean ± SEM. * *p* < 0.05; ** *p* < 0.01; *** *p* < 0.001; **** *p* < 0.0001.

The TCA cycle serves as a central hub in mitochondrial OXPHOS for the production of ATP. To elucidate whether the TCA cycle or OXPHOS affects radiotherapy sensitivity, we then generated an isogenic radioresistant cell KYSE410R (410R) by exposing KYSE410 (410) cells to gradient dose irradiation up to a total of 36 Gy (**Figure** **2**a). Consistent with the serum metabolomic signatures in nCRT pCR ESCC patients, IR‐treated parental cells exhibited accumulation of TCA cycle intermediates, whereas IR‐treated radioresistant cells showed no significant changes in these metabolites, which is aligned with the metabolomic profile of nCRT non‐pCR patients (Figure S2a, Supporting Information). Compared with 410 cells, 410R cells showed more oxygen consumption (OCR) (Figure 2b). Oxygen consumption was increased by irradiation in both 410 and 410R cells, but more dramatically in 410R cells (Figure 2b). As a consequence, ATP generation was induced by irradiation, especially in 410R cells (Figure 2c), implying that radioresistant cells might be more dependent on the TCA cycle and OXPHOS. Consistently, inhibitors blocking TCA cycle (CPI‐613 (Devimistat), fumarate hydratase‐IN‐1 (FH‐IN‐1), 3‐nitropropionic acid (3‐NP)) or OXPHOS (IACS‐010759, Rotenone, Gboxin, Antimycin A) increased the radiosensitivity of human esophageal carcinoma cells (Figure 2d,e; Figure S2b, Supporting Information). Notably, OXPHOS inhibitors blocked the entire TCA cycle, resulting in the accumulation of multiple TCA cycle intermediates, which was in line with the metabolite alterations in the serum of nCRT pCR ESCC patients (Figure 2f). However, TCA cycle inhibitor treatment significantly accumulated upstream intermediates but reduced downstream intermediates, which was not consistent with metabolite alteration in the serum of nCRT pCR ESCC patients (Figure S2c, Supporting Information). Thus, nCRT pCR ESCC patients exhibit the blockade of OXPHOS, while sustained OXPHOS activity appears to be a key determinant of nCRT resistance in ESCC.

### 2.3 AREG and EREG Regulate ESCC Radiosensitivity Through OXPHOS

**Figure 3:**
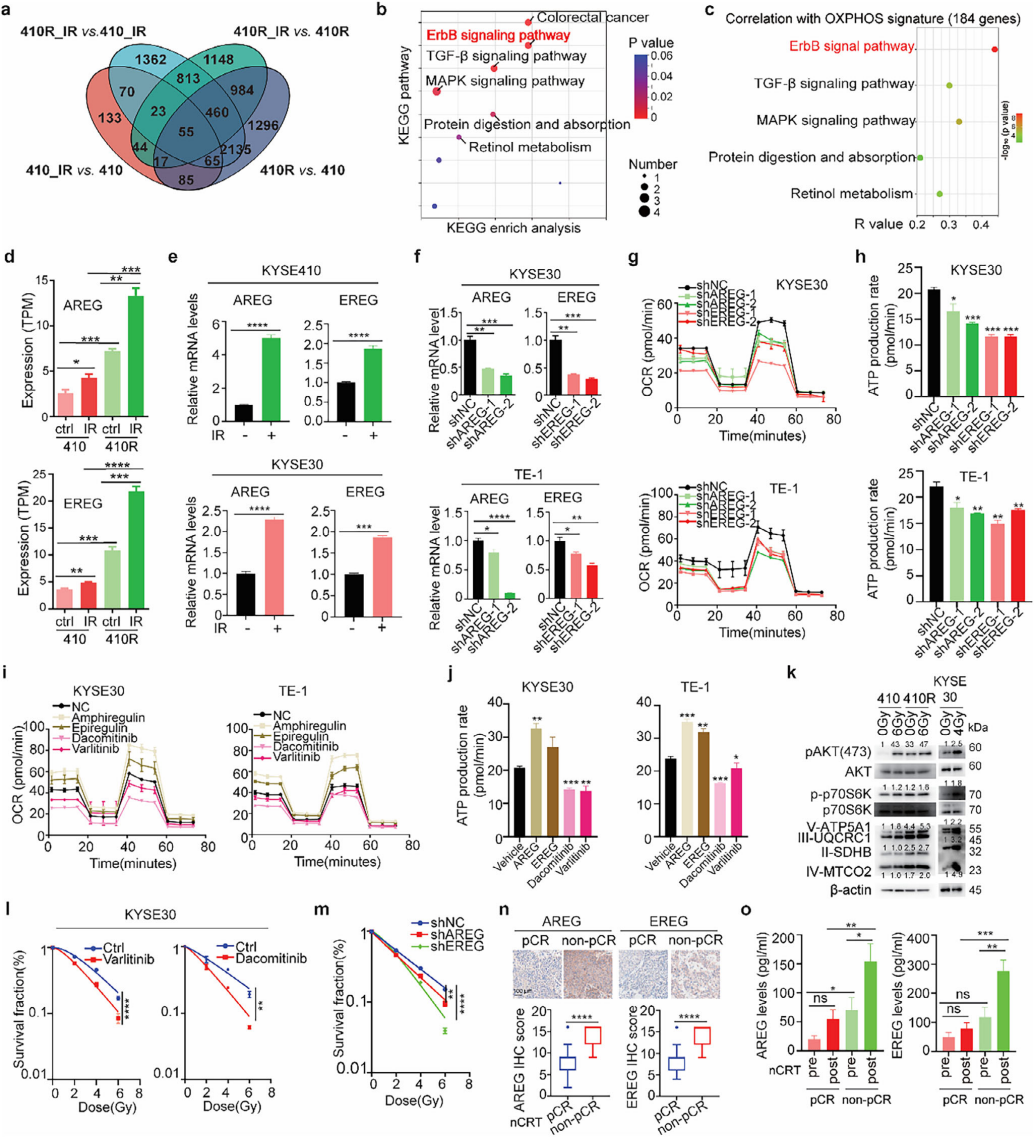
AREG and EREG modulate ESCC radiosensitivity through OXPHOS. a) Venn diagram of differentially expressed genes (DEGs) identified by RNA‐seq of KYSE410R or KYSE410 cells with or without irradiation (IR) (410R vs. 410, 410_IR vs. 410, 410R_IR vs. 410R, and 410R_IR vs. 410_IR). b) KEGG pathway enrichment of 55 overlapping DEGs from the four comparisons. c) Correlation between the expression of signature genes from DEGs‐enriched signaling pathways and OXPHOS signature gene expression in TCGA ESCA dataset. d,e) Irradiation‐induced upregulation of AREG and EREG mRNA levels was validated by RNA‐seq data (d) and qRT‐PCR (e). f–j) ErbB signaling pathway regulates OXPHOS activity. Knockdown efficacy of AREG or EREG was confirmed by qRT‐PCR (f). Oxygen consumption ratio (OCR) (g) and ATP production (h) were analyzed by Seahorse XF96 analyzer in control and AREG or EREG knockdown ESCC cells. AREG (Amphiregulin) and EREG (Eipregulin) treatment increased OCR (i) and ATP generation (j), but ERBB inhibitors (Dacomitinib or Varlitinib) treatment decreased OCR (i) and ATP generation (j) of ESCC cells. k) Irradiation resistance and IR treatment elevated mitochondrial complex proteins expression via AKT/mTOR signaling. l) ErbB inhibitors (Dacomitinib or Varlitinib) treatment increased the radiosensitivity of ESCC cells. m) Knockdown of AREG or EREG increased radiosensitivity of ESCC cells. n) IHC staining revealed higher AREG and EREG expression in nCRT non‐pCR tumor tissues versus pCR samples. o) ELISA quantification confirmed elevated baseline and nCRT‐induced serum AREG/EREG levels in non‐pCR compared to pCR ESCC patients. Two‐way ANOVA was used for statistical analyses of l and m. Student's *t*‐test was used for others. Data are presented as median with lower (Q1‐1.5*IQR) and upper (Q3 + 1.5*IQR) whiskers for n. Data are presented as mean ± SEM for others. * *p* < 0.05; ** *p* < 0.01; *** *p* < 0.001; **** *p* < 0.0001; ns, not significant.

To illustrate the underlying mechanism of how OXPHOS is regulated by radiotherapy in ESCC, the transcriptome of KYSE410 and KYSE410R cells before (pre‐) or after (post‐) irradiation (IR) was analyzed (**Figure** **3**a). The Venn diagram illustrated 55 differentially expressed genes (DEGs) in four pairwise comparisons (410R vs. 410, 410_IR vs. 410, 410R_IR vs. 410R, and 410R_IR vs. 410_IR) (Figure 3a). With KEGG pathway analysis, 55 DEGs were mainly enriched in colorectal cancer, ErbB, TGF‐β, MAPK signaling pathways (Figure 3b). Among these signaling pathways, ErbB pathway (86 signature genes) showed the strongest correlation with OXPHOS signature (184 genes) (R = 0.44, *p* = 6.8e‐10) in TCGA ESCA dataset (Figure 3c), indicating ErbB pathway might regulate OXPHOS in esophageal carcinoma. AREG and EREG, two EGF‐like growth factors that bind ERBBs to activate this pathway,^[^ ^24^, ^25^, ^26^, ^27^ ^]^ were upregulated by IR in ESCC cells, and were highly expressed in 410R cells compared with 410 cells (Figure 3d,e; Figure S3a, Supporting Information). Interestingly, OXPHOS was regulated by ErbB pathway in ESCC cells. First, knockdown of AREG or EREG significantly reduced oxygen consumption (OCR) and ATP generation in ESCC cells (Figure 3f–h; Figure S3b, Supporting Information). Second, AREG (Amphiregulin) or EREG (Epiregulin) ligands directly activated OXPHOS as they increased OCR and ATP generation (Figure 3i,j; Figure S3c, Supporting Information). Thirdly, blocking ErbB pathway by ERBB inhibitors (Varlitinib or Dacomitinib) treatment reduced OXPHOS activity as OCR and ATP generation were decreased (Figure 3i,j; Figure S3c Supporting Information). The AKT/mTOR signaling pathway, which is regulated by ERBB receptor activity, plays a crucial role in modulating the expression of mitochondrial complexes involved in OXPHOS.^[^ ^28^, ^29^ ^]^ Consistently, compared to parental KYSE410 cells, 410R exhibited significantly higher phosphorylation levels of AKT and p70S6K as well as the expression of complex II (SDHB), complex III (UQCRC1), complex IV (MTCO2), and complex V (ATP5A1) (Figure 3k). IR also activates this pathway, although the extent of pathway activation varies across different cell lines, likely due to their differential sensitivity in response to irradiation (Figure 3k; Figure S3d, Supporting Information). These data suggest that ErbB pathway regulates OXPHOS in response to IR in ESCC cells.

We further evaluated the correlation between ErbB pathway and radiosensitivity in ESCC cells. First, inhibition of ErbB pathway by varlitinib or dacomitinib sensitized ESCC cells to irradiation (Figure 3l; Figure S3e, Supporting Information). Second, knockdown of AREG or EREG increased radiosensitivity of ESCC cells (Figure 3m; Figure S3f, Supporting Information). Thirdly, AREG and EREG were highly expressed in nCRT non‐pCR compared with pCR ESCC patients (Figure 3n), suggesting that high levels of AREG or EREG are associated with nCRT resistance of ESCC patients. More importantly, AREG or EREG levels were much higher in the serum of nCRT non‐pCR than those of pCR patients, especially after nCRT (Figure 3o), indicating that AREG and EREG potentially serve as liquid biopsy‐based biomarkers for nCRT response of ESCC patients. In summary, the data suggest that AREG and EREG regulate OXPHOS of ESCC cells through the ERBB/mTOR axis, thereby impacting the radiosensitivity of ESCC cells.

### 2.4 AREG and EREG Promote Tumorigenesis in ESCC Cells

**Figure 4:**
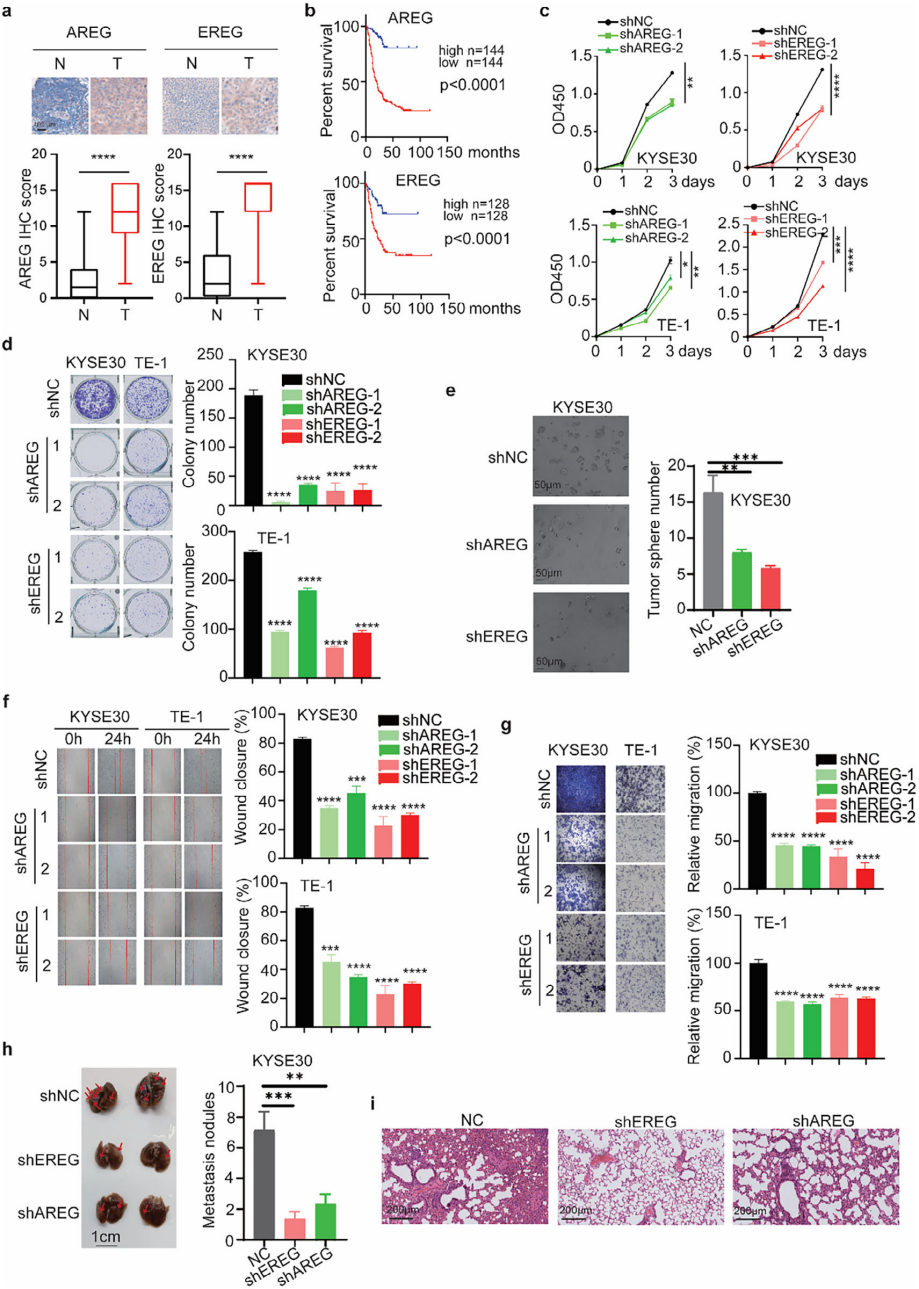
AREG and EREG promote the tumorigenesis of ESCC. a) IHC staining revealed the upregulation of AREG and EREG in ESCC tumor samples compared to normal tissues. b) High levels of AREG or EREG correlated with worse overall survival of ESCC patients. c–i) Functional validation of AREG and EREG in tumor progression. Knockdown of AREG or EREG in ESCC cells reduced the cell proliferation (c), colony formation ability (d), 3D tumor sphere formation (e), wound healing (f), migration by transwell (g), and metastasis nodules in lungs by tail vein injection in vivo (h and i). Kaplan‐Meier analysis was applied for b. Two‐way ANOVA was used for statistical analysis of c. Student's *t*‐test was used for others. Data are presented as median with lower (Q1‐1.5*IQR) and upper (Q3 + 1.5*IQR) whiskers for a. Data are presented as mean ± SEM for others. * *p* < 0.05; ** *p* < 0.01; *** *p* < 0.001; **** *p* < 0.0001.

AREG and EREG were upregulated in many cancer types, including ESCA based on TCGA database (Figure S4a, Supporting Information). Consistently, AREG and EREG were also highly expressed in ESCC tumor samples compared with adjacent normal tissue samples (**Figure** **4**a). High expression levels of AREG or EREG were correlated with worse overall survival of ESCC patients (Figure 4b) as well as many other cancer types (Figure S4b,c, Supporting Information). Knockdown of AREG or EREG with shRNAs in ESCC cells significantly suppressed their proliferation (Figure 4c), colony formation (Figure 4d), tumor sphere formation (Figure 4e), wound healing (Figure 4f), and migration (Figure 4g). In vivo, knockdown of AREG or EREG also reduced the formation of metastatic nodules in the lungs (Figure 4h,i). These data suggest that AREG and EREG are oncogenes in ESCC.

### 2.5 CEBPB Regulates the Expression of AREG and EREG in Response to Irradiation in ESCC Cells

**Figure 5:**
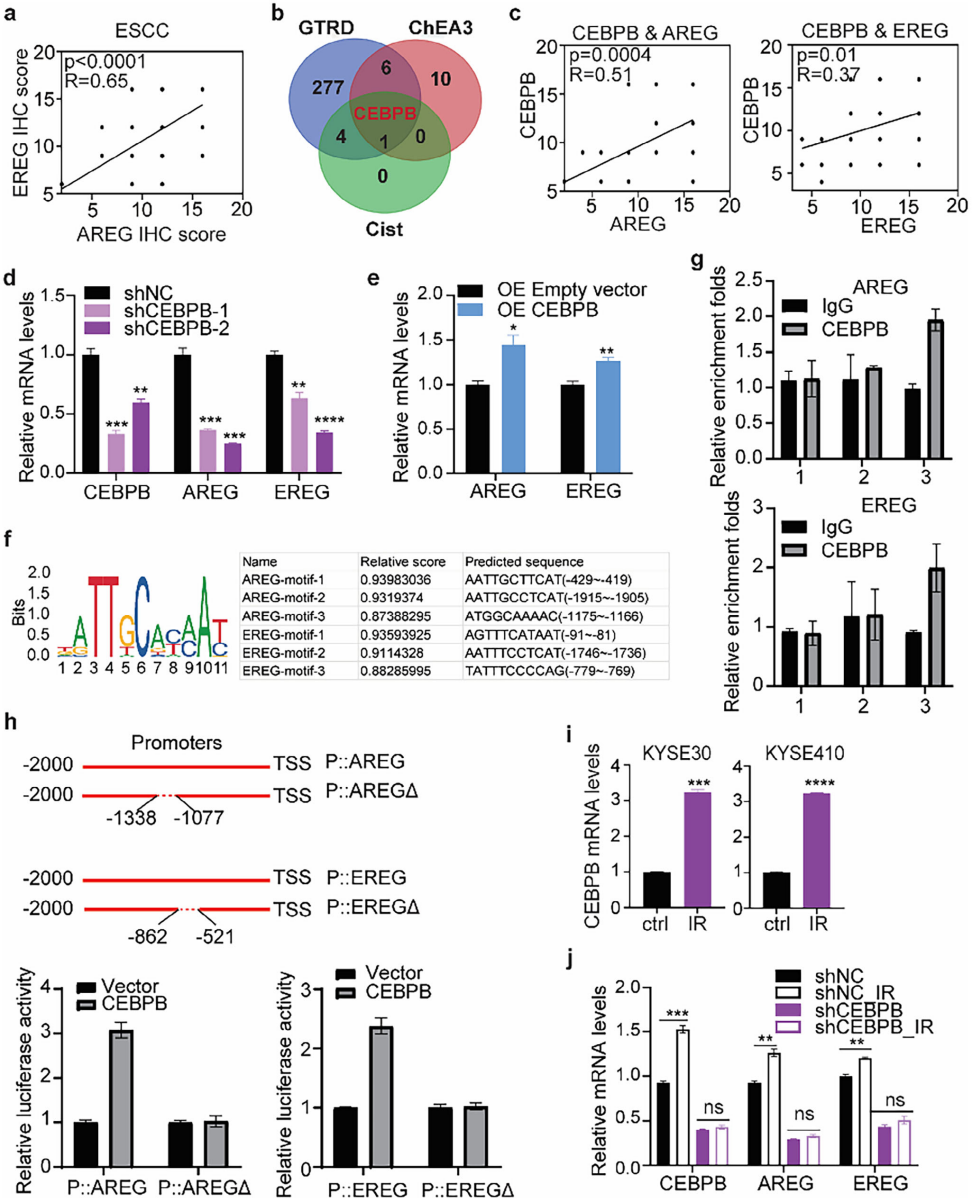
CEBPB acts as a co‐transcription factor for AREG and EREG in response to irradiation. a) IHC staining revealed significant co‐expression of AREG and EREG in ESCC tumor samples (*N* = 45). b) Venn diagram identifying shared transcriptional regulators of AREG and EREG predicted by CISTROME, ChEA3, and GTRD databases. c) IHC staining revealed a positive correlation between CEBPB and AREG or EREG protein levels in ESCC tumor samples (*N* = 45). d–h) CEBPB is a transcription factor of AREG and EREG. CEBPB knockdown attenuated AREG and EREG mRNA levels (d), and CEBPB overexpression elevated AREG and EREG transcription (e). CEBPB binding motifs were predicted by JASPAR (https://jaspar.elixir.no/) (f). ChIP‐PCR was performed to validate the CEBPB binding motif on promoters of AREG and EREG (g). Luciferase assay was performed to determine the direct activation of CEBPB on AREG and EREG (h). i) Irradiation (IR) induced CEBPB transcription. j) IR‐induced AREG and EREG expression were abrogated by knockdown of CEBPB. Student's *t*‐test was applied for statistical analyses. Data are presented as mean ± SEM. * *p* < 0.05; ** *p* < 0.01; *** *p* < 0.001; **** *p* < 0.001; n.s., not significant.

Interestingly, the mRNA levels of AREG and EREG were highly correlated in almost all cancer types (Figure S5a, Supporting Information). In ESCC tumor samples, the expression of AREG and EREG was also highly correlated (**Figure** **5**a), suggesting that AREG and EREG are commonly regulated simultaneously. Since both AREG and EREG were upregulated by IR, we assumed that they share the same transcription factor, which is regulated by IR. Three different online software (CIST, GTRD, and ChEA3) were applied to predict the common transcription factors for AREG and EREG, and integrated analyses showed that CEBPB (CCAAT Enhancer Binding Protein Beta) was the only transcription factor to co‐regulate AREG and EREG (Figure 5b). The expression of CEBPB was correlated with the expression of AREG or EREG in ESCC tumor samples and the TCGA ESCA dataset (Figure 5c; Figure S5b, Supporting Information). Knockdown of CEBPB reduced the expression of AREG and EREG (Figure 5d), while overexpression of CEBPB induced their expression in ESCC cells (Figure 5e; Figure S5c, Supporting Information). With the ChIP‐PCR assay, we confirmed the binding region of CEBPB on the promoters of AREG or EREG (Figure 5f,g). CEBPB directly activated the transcription of AREG or EREG, which were abolished by deletion of the CEBPB binding region on their promoters (Figure 5h). CEBPB was also upregulated in response to irradiation as AREG and EREG (Figure 5i). More importantly, knockdown of CEBPB significantly abolished the induction of AREG and EREG by irradiation (Figure 5j), suggesting that AREG and EREG are regulated by CEBPB in response to radiation in ESCC cells. Interestingly, the CEBPB/AREG/EREG axis was responsive to irradiation in various cancer cells but not in noncancerous cells (Figure S5d–f, Supporting Information).

Epigenetic mechanisms such as DNA methylation and histone methylation have also been reported to respond to radiotherapy.^[^ ^30^, ^31^, ^32^, ^33^ ^]^ However, in ESCC cells, we observed no significant alterations in DNA methylation levels at the promoter regions of AREG, EREG, or CEBPB or histone methylation (H3K4me3, H3K27me3, and H3K36me3) following irradiation (Figure S5g,h, Supporting Information), suggesting that the radiation‐induced upregulation of AREG and EREG is likely mediated through CEBPB, rather than via these epigenetic mechanisms.

### 2.6 CEBPB Regulates ESCC Radiosensitivity Through OXPHOS

**Figure 6:**
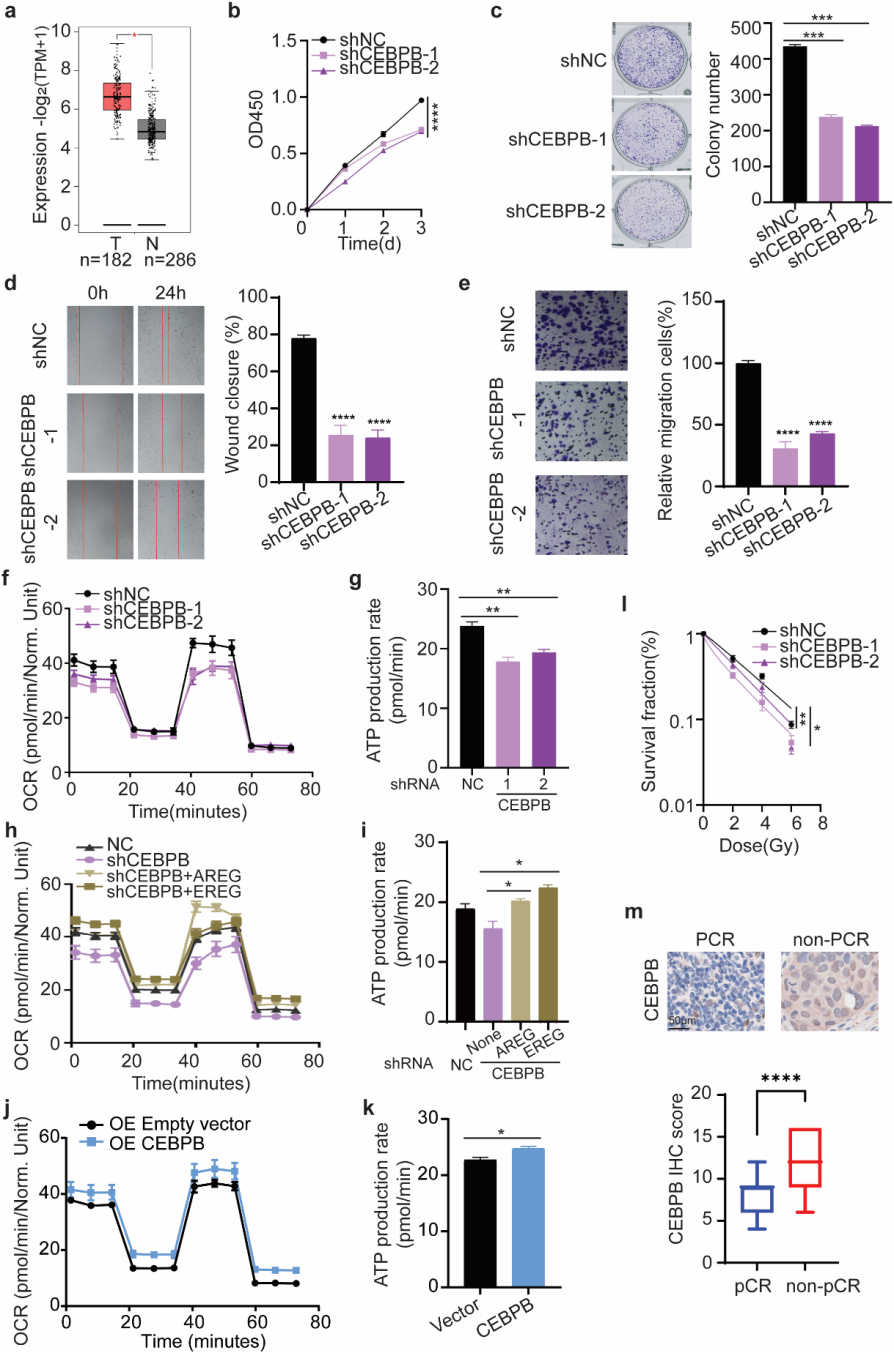
CEBPB drives tumor progression and OXPHOS‐mediated radioresistance in ESCC. a) CEBPB expression was elevated in ESCA tumor samples compared to normal tissue samples (GEPIA). b–e) CEBPB promotes the tumorigenesis of ESCC. CEBPB knockdown in KYSE30 cells reduced the cell proliferation (b), colony formation ability (c), wound healing (d), and migration by transwell (e). f–i) CEBPB regulates OXPHOS activity through AREG and EREG. Knockdown of CEBPB reduced oxygen consumption ratio (OCR) (f) and ATP production (g). The reduction of OCR (h) and ATP generation (i) in CEBPB knockdown cells can be rescued by adding exogenous AREG or EREG. Overexpression of CEBPB increased OCR (j) and ATP generation (k). l Knockdown of CEBPB increased radiosensitivity of KYSE30 cells. m) IHC revealed higher CEBPB protein levels in nCRT non‐pCR ESCC tumors versus pCR specimens. Student's *t*‐test was applied for statistical analyses. Data are presented as median with lower (Q1‐1.5*IQR) and upper (Q3+1.5*IQR) whiskers for k. Data are presented as mean ± SEM for others. * *p* < 0.05; ** *p* < 0.01; *** *p* < 0.001; **** *p* < 0.0001.

CEBPB was upregulated in the TCGA ESCA database (**Figure** **6**a). Knockdown of CEBPB reduced the proliferation, colony formation, wound healing, and migration of ESCC cells (Figure 6b–e), just as knockdown of AREG or EREG, indicating the oncogenic role of CEBPB in ESCC cells. Strikingly, knockdown of CEBPB also reduced oxygen consumption and ATP generation in ESCC cells (Figure 6f,g; Figure S6a, Supporting Information), which can be rescued by adding exogenous AREG or EREG (Figure 6h,i). Overexpression of CEBPB increased their oxygen consumption and ATP generation (Figure 6j,k; Figure S6b, Supporting Information). Meanwhile, knockdown of CEBPB increased radiosensitivity of ESCC cells (Figure 6l; Figure S6c, Supporting Information). In terms of molecular mechanism, knockdown of AREG, EREG, or CEBPB blocked the IR‐induced phosphorylation of AKT and p‐p70S6K as well as the expression of several subunits of mitochondrial complex (Figure S6d, Supporting Information), indicating that CEBPB/AREG/EREG mediates the activation of AKT/mTOR/OXPHOS in response to irradiation. Moreover, the expression of CEBPB was higher in nCRT non‐pCR compared with nCRT pCR ESCC patients (Figure 6m). The data suggest that CEBPB, the transcription factor of AREG and EREG, regulates radiosensitivity of ESCC cells through OXPHOS.

We further investigated whether AREG, EREG, or CEBPB regulates radiosensitivity through mechanisms beyond OXPHOS. Irradiation activated DNA damage repair (elevated p‐ATM and p‐ATR levels), which was abolished by knockdown of AREG, EREG, or CEBPB (Figure S6e, Supporting Information), suggesting that the CEBPB/AREG/EREG axis may simultaneously regulate both DNA damage repair and OXPHOS to influence ESCC radiosensitivity.

### 2.7 Inhibition of OXPHOS or AREG/EREG/ERBB Improves Radiosensitivity of ESCC Tumors

**Figure 7:**
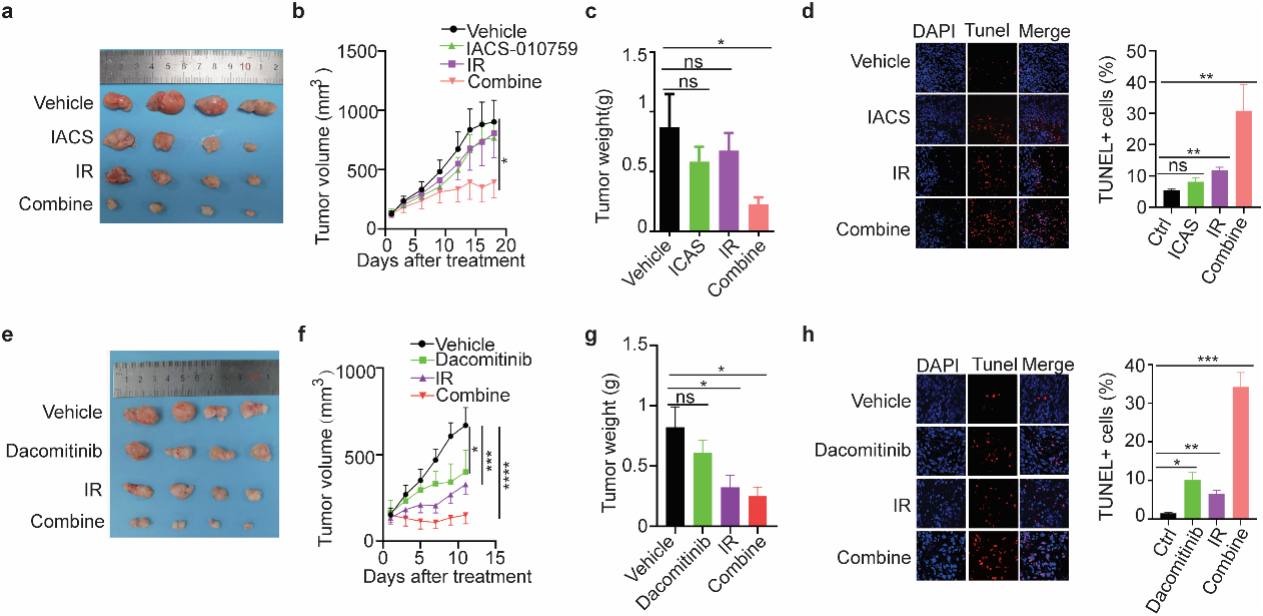
Targeting OXPHOS or ERBB enhances radiosensitivity of ESCC tumors. a–d) IACS‐010759 (Mitochondrial complex I inhibitor) enhances irradiation efficacy for ESCC xenograft tumors. Mice harboring KYSE30‐derived xenograft tumors were divided into four groups: vehicle (PBS) (*N* = 9), IACS‐010759 (7.5 mg kg^−1^) (*N* = 10), irradiation (10 Gy) (*N* = 7), and combination of IACS‐010759 and irradiation (*N* = 10). Xenograft tumors were presented in (a). Tumor volumes (b) and tumor weights (c) of xenograft tumors were measured at the indicated time points. Apoptotic cells in xenograft tumors were detected by TUNEL assay (d). e–h) Dacomitinib (pan‐ERBB inhibitor) enhances irradiation efficacy for ESCC xenograft tumors. Mice harboring KYSE30‐derived xenograft tumors were divided into four groups: vehicle (PBS) (*N* = 9), dacomitinib (7 mg kg^−1^) (*N* = 7), irradiation (10 Gy) (*N* = 7), and combination (*N* = 6). Xenograft tumors were presented in (e). Tumor volumes (f) and tumor weights (g) of xenograft tumors were measured at the indicated time points. Apoptotic cells in xenograft tumors were detected by TUNEL assay (h). Irradiation with a single dose at 10 Gy. Two days later, oral gavage was applied for PBS vehicle, IACS‐010759 (7.5 mg kg^−1^) or dacomitinib (7 mg kg^−1^) thrice a week. Two‐way ANOVA was used for statistical analyses of b and f. Student's *t*‐test was used for others. Data are presented as mean ± SEM. * *p* < 0.05; ** *p* < 0.01; *** *p* < 0.001; **** *p* < 0.0001; ns, not significant.

As the AREB/EREG/ERBB axis regulates ESCC radiosensitivity through OXPHOS, inhibition of OXPHOS or the ErbB signaling pathway may be a good strategy to enhance the radiotherapy effect. Inhibitors targeting OXPHOS complex I (IACS‐010759) or targeting ERBBs (dacomitinib) are now under evaluation in clinical trials for cancer therapy. IACS‐010759 or IR alone showed moderate inhibition for subcutaneous xenograft tumors generated by KYSE30 cells, however, the combination of IACS‐010759 and IR significantly inhibited the growth of these tumors (**Figure** **7**a–c). TUNEL assay showed that the combination of IACS‐010759 and IR induced many more apoptotic cells than either IR or IACS‐010759 alone (Figure 7d). Similarly, dacomitinib largely improved the radiosensitivity of subcutaneous xenograft tumors (Figure 7e–g), and the combination of dacomitinib and IR induced much more cell death than either IR or dacomitinib alone (Figure 7h). Moreover, the body weights of mice were not dramatically affected by such combinations (Figure S7, Supporting Information). In summary, either inhibition of OXPHOS or ERBB could enhance the radiosensitivity for ESCC tumors.

## 3 Discussion

We demonstrate that OXPHOS plays pivotal role in radioresistant cells and ESCC patients. Actually, several studies have shown the effect of OXPHOS on radiotherapy sensitivity. OXPHOS inhibitors such as metformin or phenformin enhance the sensitivity of tumors to irradiation.^[^ ^34^, ^35^ ^]^ Knockdown of isocitrate dehydrogenase 2 increases radiosensitivity of ESCC cells.^[^ ^13^ ^]^ These findings all support the conclusion that OXPHOS activity could be an important for nCRT sensitivity. Interestingly, several studies reported inhibitors targeting glycolysis such as HK2 or PDK1 also sensitize cancer cells to irradiation.^[^ ^36^, ^37^, ^38^, ^39^ ^]^ It is possible that glycolysis inhibitors also have influence on OXPHOS thereby increasing radiosensitivity of cancer cells.

Beyond the current study, other metabolomics‐based screening investigations have revealed the enrichment of TCA cycle intermediates (e.g., citrate, isocitrate) in the plasma of patients who were sensitive to radiotherapy or neoadjuvant chemoradiotherapy.^[^ ^16^ ^]^ This suggests that OXPHOS blockade may represent a metabolic hallmark of radiotherapy‐sensitive individuals.^[^ ^40^ ^]^ TCA cycle intermediates accumulation may directly be attributed to their release from local dying cancer cells or OXPHOS blockade in residual living cells. On the other hand, radiotherapy can provoke profound systemic immune alterations that indirectly interfere OXPHOS in various tissues. The abscopal effect, a phenomenon where local irradiation induces immune‐mediated regression of non‐irradiated metastases, underscores this systemic response.^[^ ^41^ ^]^ The inflammatory cytokines (e.g., TNF‐α, IFN‐γ, IL‐6, etc) released during this systemic immune response interfere with mitochondrial function or regulate the export of TCA cycle intermediates in distant tissues, potentially resulting in TCA cycle intermediates accumulation in serum.^[^ ^42^, ^43^, ^44^, ^45^ ^]^ Establishing orthotopic ESCC mouse models for localized radiotherapy may help clarify whether changes in TCA cycle metabolites originate primarily from the tumor itself or from normal tissues.

How OXPHOS was regulated in response to IR in ESCC is largely unknown. This study reveals that two ERBB ligands, AREG and EREG, which are highly expressed in ESCC tumor samples, have shown an impact on OXPHOS through the ERBB/mTOR axis, thereby conferring radioresistance of ESCC cells. AREG modulates radiosensitivity of colorectal cancer cells through the PI3K/AKT axis.^[^ ^46^ ^]^ Targeting its receptor EGFR/ERBB1 enhances radiosensitivity in several cancer types.^[^ ^47^, ^48^, ^49^, ^50^ ^]^ Targeting HER2/ERBB2 combined with radiotherapy also shows great potential in breast cancer treatment.^[^ ^51^, ^52^ ^]^ Intriguingly, beyond their established roles in distinct signaling cascades modulating radiosensitivity, these molecules may exhibit co‐dependence on a metabolic hub, OXPHOS, to control radioresponse in ESCC. More importantly, as secret EGF‐like growth factors, we also proved that AREG and EREG can serve as liquid biopsy‐based biomarkers to monitor nCRT response of ESCC patients. Large‐scale population cohort studies are essential to validate the clinical value of AREG or EREG as prognostic biomarkers for nCRT of ESCC patients in the future.

The regulatory mechanisms of AREG and EREG gene expressions in ESCC are poorly understood. It has been demonstrated that AP‐1, STAT3, EGFR/MAPK, or Wnt/β‐catenin pathways can regulate the expression of AREG or EREG across malignancies.^[^ ^53^, ^54^, ^55^ ^]^ Interestingly, the expression levels of AREG and EREG are well‐correlated in many cancers, including ESCC, which leading us to elucidate the underlying mechanism co‐regulating AREG and EREG simultaneously. We unveil CEBPB as co‐transcription factor of both AREG and EREG in response to irradiation in ESCC cells. With public datasets, irradiation generally induced high expression of AREG in various cancer cells, and co‐upregulation of EREG or CEBPB was observed in some cancer cells. These findings suggest that CEBPB/AREG/EREG may play broad roles in mediating radiotherapy responses across multiple cancers. However, in normal non‐carcinoma cells, the expression of AREG, EREG, and CEBPB remained largely unaltered or was even reduced following radiotherapy, supporting the notion that the CEBPB/AREG/EREG axis operates through a cancer‐specific mechanism in response to irradiation.

CEBPB is a transcription factor regulating the expression of genes involved in immune and inflammatory responses.^[^ ^56^ ^]^ CEBPB has been reported to promote tumorigenesis through STAT3, PI3K/AKT, or EMT signaling pathways in breast cancer, renal cancer, or colorectal cancer.^[^ ^57^, ^58^, ^59^ ^]^ We demonstrated that CEBPB, as well as its target genes AREG and EREG, are critical for the tumorigenesis of ESCC, which is in line with their function in other cancers.^[^ ^60^ ^]^ NRF2, a transcriptional factor that responds to irradiation,^[^ ^61^, ^62^ ^]^ has been shown to regulate CEBPB in adipogenesis.^[^ ^63^ ^]^ Whether irradiation may modulate CEBPB through NRF2 in ESCC is worth further investigation.

Since ERBB could regulate radioresistance through OXPHOS for ESCC cells, targeting mitochondrial OXPHOS or ERBB may be good strategies to enhance the irradiation efficacy. Not like metformin or phenformin, IACS‐0101759 is an extremely specific inhibitor of mitochondrial complex I under clinical trials for cancer treatment.^[^ ^64^ ^]^ Consistent with our hypothesis, IACS‐010759 significantly improved the radiosensitivity of ESCC xenograft tumors. To inhibit receptors of both AREG (ERBB1 ligand) and EREG (ERBB1/ERBB4 ligand), we selected a pan‐ERBBs inhibitor dacomitinib^[^ ^65^ ^]^ and found that dacomitinib also significantly suppressed ESCC xenograft tumors' growth when it was combined with IR. Therefore, our study found that targeting ERBB or OXPHOS can effectively enhance radiotherapy sensitivity, providing a theoretical basis for the treatment strategy of ESCC.

## 4 Experimental Section

### Human Tissues Collection

This study received approval from the Ethics Committee at Renji Hospital, Shanghai Jiao Tong University School of Medicine (Shanghai, China) (KY2020‐032). Informed consent was obtained from all participants. Eligible patients were pathologically confirmed locally advanced resectable thoracic ESCC, stage T1‐4aN1‐3M0 or T3‐4aN0M0 (stages II–IVA) according to the eighth edition of the American Joint Committee on Cancer staging manual. All patients received nCRT at Renji Hospital from 2020 to 2022. A total dose of 41.4 Gy was administered in 23 fractions, on five fractions per week. The chemotherapy regimen consisted of paclitaxel (45 mg m^−2^) and cisplatin (25 mg m^−2^), which were administered weekly on days 1, 8, 15, 22, and 29. Surgery was scheduled for 4 to 6 weeks after the course of nCRT. The response to nCRT was evaluated based on the pathological results from surgical specimens. They were classified into two groups: pathological complete response (pCR) and non‐pathological complete response (non‐pCR). The baseline clinical features for patients with pCR (*N* = 23) and non‐pCR (*N* = 36) were presented in Table S1 (Supporting Information). Serum samples were collected in the morning following an overnight fast, just before the initiation of nCRT, and after the last day of nCRT. All samples were stored at −80 °C for subsequent metabolome analysis, including untargeted and targeted detections.

### Cell Culture

ESCC cell lines, including KYSE30, KYSE410, TE‐1 were purchased from American Type Culture Collection. KYSE410 and TE‐1 were maintained in RPMI 1640 medium (Sigma‐Aldrich, USA). KYSE30 was maintained in the medium including 50% RPMI 1640, 50% Ham's F‐12, and 2 mmol L^−1^ L‐glutamine. All cell lines were maintained at 37 °C in a 5% CO_2_ atmosphere with medium supplemented with 10% fetal bovine serum (FBS, Yeasen, China) and 1% penicillin‐streptomycin (Gibco, USA). Cell culture medium was routinely assayed for mycoplasma to ensure that they were mycoplasma‐free (Yeasen).

15000 cells were seeded in MatrigelTM (15 µL) (Corning, USA) per well in 48‐well plates to do 3D culture. It was fed with Advanced DMEM/F12 (250 µL) (Gibco, USA) supplemented with HEPES (10 mmol L^−1^) (Gibco, USA), 1× Glutamax (gibco, USA), Nicotinamide (10 mmol L^−1^) (Sigma‐Aldrich, USA), N‐acetyl‐L‐cysteine (1 mmol L^−1^) (Sigma‐Aldrich, USA), Y‐27632 (10 µmol L^−1^) (Abmole, USA), A83‐01 (500 nmol L^−1^) (Yeasen, China), 1× N2 (Yeasen, China), 1× B27 (Yeasen, China), mouse recombinant epidermal growth factor (50 ng mL^−1^) (Yeasen, China), and Noggin/R‐Spondin‐conditioned medium (100 ng mL^−1^) (Yeasen, China). Then incubate at 37 °C with 5% CO2 for 2 days in a humidified incubator.

### Reagents

HPLC‐grade methanol, acetonitrile, and formic acid were purchased from Thermo Fisher Scientific (Waltham, MA, USA). 2‐chloro‐L‐phenylalanine was purchased from Sigma Aldrich (MO, USA). Ultrapure water was produced by a Mill‐Q Reference system equipped with a LC‐MS Pak filter (Millipore, MA, USA).

Rotenone was purchased from Sigma (St. Louis, USA). Amphiregulin, dacomitinib, epiregulin, varlitinib, FH‐IN‐1, IACS‐010759, and MDH1‐IN‐1 were purchased from MedChemExpress (Shanghai, China). CPI‐613, Gboxin, and 3‐nitropropionic acid (3‐NP) were purchased from Selleck (Shanghai, China). Antiymcin A was purchased from Glpbio (California, USA). For the in vivo experiment, IASC‐010759 was purchased from CSNpharm (Shanghai, China). Dacomitinib was purchased from Macklin (Shanghai, China).

### Untargeted Metabolomic Profiling

Serum samples from ESCC patients were freely thawed at 4 °C for 30 min. For each sample, pre‐chilled methanol (300 µL, containing 2‐chloro‐L‐phenylalanine (2 µg mL^−1^) as internal standard) was added to serum (100 µL) and mixed for 15 s on a vortex. Then samples were centrifuged at 12 000 rpm for 15 min at 4 °C to remove protein precipitation. After centrifugation, the supernatants were dried with nitrogen gas. The dried samples were reconstituted in 80% methanol (80 µL). The constitution was then centrifuged at 12 000 rpm for 15 min at 4 °C, and supernatant (50 µL) was transferred to a fresh glass vial for untargeted detection in UHPLC‐QTOF MS (AB sciex) and targeted detection in UHPLC‐QTRAP MS (AB sciex). The untargeted detection for LC separation was carried out using a 1290 Infinity series UHPLC System (Agilent Technologies), equipped with a Waters ACQUITY UPLC BEH Amide column (1.7 µm, 2.1 mm×100 mm). The mobile phase A was 0.1% formic acid in water, and B was acetonitrile. The analysis was carried out with elution gradient as follows: 0–0.5 min, 95% B; 0.5–7.0 min, 95%–65% B; 7.0–8.0 min, 65%–40% B; 8.0–9.0 min, 40% B; 9.0–9.1 min, 40%–95% B; 9.1–12.0 min, 95% B. The column temperature was 25 °C. The auto‐sampler temperature was set at 4 °C, and the injection volume was 2 µL.

The TripleTOF 6500 mass spectrometry (AB Sciex) was used to acquire MS/MS spectra on an information‐dependent acquisition (IDA) mode. Sample detection was performed in the negative mode of electrospray ionization (ESI) source. In each cycle, the most intensive 12 precursor ions with intensity above 100 were chosen for MS/MS at a collision energy (CE) of 30 eV. The cycle time was 0.56 s. ESI source conditions were set as follows: Gas 1 as 60 psi, Gas 2 as 60 psi, Curtain Gas as 35 psi, Source Temperature as 550 °C, Declustering potential as 60 V. After data acquisition, all the MS raw data (.wiff) files were converted to the mzXML format by ProteoWizard, and processed by XCMS^plus^ (version 3‐6‐14). The process includes feature detection, retention time alignment, grouping, and integration. Feature detection (centWave) was set as PPM = 20, Minimum Peak Width = 5, Maximum Peak Width = 40, m/z diff = 0.01. Batch correction was realized by retention time alignment (obiwarp, binSize = 0.1). Grouping was set as Minfrac = 0.5, mzwid = 0.015, minsamp = 0.5, max = 50. The missing values were replaced with the half minimum. The area for each peak was normalized to the internal standard (2‐chloro‐L‐phenylalanine). Then the dataset was imported into SIMCA‐P (16.0) to perform multivariate analysis, including PCA and PLS‐DA. Finally, peaks satisfied with VIP>1.2 (VIP = variable importance in the projection) were filtered as differentiated ions. Metabolites identification was performed based on the local library of Accurate Mass Metabolite Spectral Library Search in the MasterView 1.1 Software (AB Sciex).

### Targeted Metabolomic Analysis

For the cell sample, 3 × 10^6^ cells were plated in 10 cm dishes. When the confluence reached 90% confluency, cells were washed with PBS twice. Cells were then quenched with 1 mL pre‐chilled methanol containing 2‐chloro‐L‐phenylalanine (2 µg mL^−1^) as internal standard, and metabolites were extracted by sonication. After removing the debris by centrifugation at 12,000 rpm for 15 min, the supernatants were transferred to a fresh glass vial for targeted detection with an Agilent 1290 UHPLC system coupled to an AB QTRAP 6500 mass spectrometry (AB Sciex, USA). A Waters ACQUITY UPLC BEH Amide column (1.7 µm, 2.1 mm × 100 mm) was used for metabolites separation. The mass spectrometer was detected in multiple reaction monitoring (MRM) mode. The MRM transitions (m/z), DPs (V), and CEs (V) of targeted metabolites for cancer metabolism were referred to previous literature.^[^ ^66^ ^]^ MRM data were acquired using Analyst 1.6.1 software (AB Sciex). Chromatogram review and peak area integration were conducted using OS software (Version 1.5.0.23389, AB Sciex). The abundances of each metabolite were presented as the peak area ratio of targeted metabolites versus internal standard 2‐chloro‐L‐phenylalanine for each sample. Metabolites satisfied with *p* < 0.05 (Student's *t*‐test) were regarded as significantly different.

### Development of Radioresistance Subclone KYSE410R Cells

KYSE410 cells were plated in 25 cm^2^ culture flasks. Cells were irradiated with 2 Gy of X‐ray using a high‐energy linear accelerator (Elekta Synergy, 6 MV X‐rays, 600 MU min^−1^). Immediately after irradiation, the culture medium was renewed, and then the cells were returned to the incubator. When they reached ≈90% confluence, the cells were trypsinized and subcultured into new flasks. When they reached ≈50% confluence, the cells were again irradiated. These procedures were repeated 18 times twice a week to a dose of 36 Gy for 2 months until radioresistant cell populations were established. Clonogenic assays were used to determine the resistance level among those clones. The parental cells were trypsinized, counted, and passaged under the same conditions without irradiation.

### Cell and Tumor Irradiation

Cells were irradiated by a medical linear accelerator (Elekta Synergy, 6 MV X‐rays, 600 MU min^−1^) with 180° fields and a field size of 40 cm × 40 cm. For in vivo, mice were irradiated with a total dose of 10 Gy (6 MV X‐rays, 600 MU min^−1^) using a medical linear accelerator (Elekta Synergy).

### Cell Transfection

To knockdown AREG/EREG/CEBPB in ESCC cells, two independent shRNAs sequences were designed. Another shRNA with a non‐targeting sequence was used as a negative control (NC). Cells were plated in 6‐well plates and transfected with lentivirus, and stable cells were screened with puromycin (Invitrogen, 4 µg mL^−1^ for KYSE30, 1.0 µg mL^−1^ for TE‐1) for 2 weeks. The efficacy for AREG/EREG/CEBPB knockdown was validated by quantitative real‐time PCR.

### Western Blot

Cells were washed three times with cold phosphate‐buffered saline (PBS), and total cellular protein was extracted using RIPA lysis buffer (Qiagen, Germany) supplemented with proteinase inhibitor cocktail and phosphatase inhibitor (Roche Applied Science, Switzerland). The lysates were incubated on ice for 30 min followed by centrifugation at 4 °C, 12 000 rpm for 15 min. Protein concentrations were analyzed using the Bicinchoninic Acid Kit (Pierce, Rockford, IL). Total proteins (40 µg) were separated by 10% Sodium dodecyl sulfate – polyacrylamide gel electrophoresis (SDS‐PAGE) and transferred onto 0.22 µm polyvinylidene fluoride membrane (PDVF; Milli pore). The membranes were blocked with 5% non‐fat dried milk for an hour at room temperature, and then incubated with primary antibodies overnight at 4 °C. After washing, the membranes were incubated with appropriate horseradish peroxidase (HRP)‐conjugated secondary antibodies. The antibodies used in this study were listed in Table S6 (Supporting Information). Protein bands were visualized by the enhanced chemiluminescence (ECL) detection kit (Tanon, China). Quantification of Western blots was analyzed by Image J.

### Immunohistochemistry Staining

Paraffin‐embedded mouse or human tissues were completely deparaffinized in xylene, and the antigen was retrieved by boiling the sample for 20 min (Beyotime Biotechnology). Samples were incubated with primary antibodies at 4 °C overnight and followed by the Immunohistochemistry Application Solutions Kit (Cell Signaling Technology) according to the manufacturer's instructions. Anti‐AREG (Proteintech, 1:400 dilution, cat. no.160‐36‐1‐AP), anti‐EREG (Absin, 1:200 dilution, cat. no. abs106776), and anti CEBPB (Proteintech, 1:400 dilution, cat. no. 23431‐1‐AP) were used in the study. Wherever the IHC scores were plotted, the staining score was based on the staining strength and the number of positive cells. The positive staining rate was scored as: 0 points for less than 5%, 1 point for 5–25%, 2 points for 26–50%, 3 points for 51–75%, and 4 points for over 75%. The staining strength was scored as: non‐staining is 0 points, light yellow is 1 point, brown‐yellow is 2 points, and brown is 3 points. The final score is the multiplication of two scores and then classified into 4 grades: negative (0 points), weakly positive (1–4 points), positive (5–8 points), and strongly positive (9‐12 points).

### Quantitative Real‐Time PCR

Total RNA was extracted from cell lines using RNAfast200 kits (Fasted, Shanghai, China) according to the manufacturer's instructions, and the quality of isolated RNAs was determined by Nanodrop (Thermo Scientific, Massachusetts, USA). Reverse transcription (RT) was conducted by PrimeScript RT Master Mix (TaKaRa, Kusatsu, Japan). cDNAs were used as templates for PCR. Real‐time PCR was carried out using ChamQTM SYBR Color qPCR Master Mix (Vazyme, Nanjing, China). ATCB (β‐actin) was applied to normalize genes’ expression. The primers and oligos used in this study were showed in Table S7 (Supporting Information).

### Proliferation and Migration Analysis

CCK8 assay was applied to evaluate the proliferation capacity of ESCC cells. In brief, 1 × 10^3^ cells were plated in each well of 96 well plates. To evaluate the proliferation capacity at 0, 24, 48, and 72 h, 10 µL CCK8 reagent was added to each well and incubated with ESCC cells for 2 h. The absorbance of ESCC cells in each well was measured by a microplate reader. To explore the migration ability, RPMI 1640 (200 µL) without serum was used to resuspend ESCC cells, and added to them in upper chamber of the Transwell chamber (Corning, Inc. USA) with 1 × 10^4^ cells/well. Subsequently, 800 µL complete medium was added to the lower chamber as the chemical attractants. After incubation for 24 h at 37 °C, cells on the lower surface of the non‐coated membrane were fixed by 4% paraformaldehyde for 10 min at room temperature and then stained by crystal violet for 30 min at room temperature. Images from five representative fields of each membrane were taken by using a light microscope (10×). The number of migratory cells was counted, and the relative migration rate was calculated.

### Wound Healing Assay

The KYSE30 and TE1 cells were seeded into a 6‐well plate, and when the cell density reached 80%, a sterile 200 µL pipette tip was used to make a straight scratch line on the confluent cell monolayer. At 0, 24, and 48 h after scratch, cell migration was imaged by a light microscope (10×). Image J was used to calculate the closure.

### Colony Formation Assay

Cells (500–3000 cells per well) were seeded into 6‐well plates and maintained at 37 °C with 5% CO_2_. Then cells were irradiated with an indicated of 0, 2, 4, 6 Gy using 6 Mv X‐rays. After 14 days of incubation, the cells were rinsed with PBS, then stained with 0.5% crystal violet. The number of colonies was counted under a microscope.

### Oxygen Consumption Rate (OCR) Analysis

OCR was measured using a Seahorse XF96 analyzer (Agilent, USA). Briefly, KYSE30 and TE1 cells were seeded in XF96 well microplates (10000 cells/well) (Agilent Technologies, Sana Clara, USA) for 24 h. For OCR measurement, cells were covered with assay medium (80 µL) (XF base medium (Seahorse, 102353), 100 mmol L^−1^ sodium pyruvate, 200 mmol L^−1^ L‐glutamine, and 1 mol L^−1^ glucose) and pre‐incubated for 45 min – 1 h in a non‐CO_2_ incubator set to 37 °C. Injection ports were performed with oligomycin (1.5 µmol L^−1^), FCCP (1 µmol L^−1^), rotenone (0.5 µmol L^−1^), and Antimycin A (0.5 µmol L^−1^), which were all provided as lyophilized powders in Mito Stress Test Kit (Agilent, USA). OCR was measured in real‐time with the Mito Stress Test Kit using Seahorse XFe96 Analyzer (Agilent, USA) following the manufacturer's instructions.

### RNA Sequencing and Data Analysis

KYSE410 and KYSE410R cells were both exposed to irradiation for 4 Gy. After 24 h, the irradiated cells (KYSE410_IR & KYSE410R_IR) together with non‐irradiated (KYSE410 & KYSE410R) were all collected. Total RNA, which was extracted via TRIzoI Reagent and quantified by NanoDrop ND‐3000 (NanoDrop Technologies), following manufacturer recommendations. High‐quality RNA was used for library construction, RNA purification, reverse transcription, and sequencing at Shanghai Majorbio Biopharm Co., Ltd (Shanghai, China), following Illumina's instructions. DEGs were identified via DESeq2, with |log2FC| ≥ 1.2 and *p* adjust < 0.05 considered significant. KEGG pathway analyses were performed following the instructions.

### Tumor Xenograft Models

A subcutaneous xenograft mouse model was used to assess tumor growth. Animal experiments were approved by the Ethics Committee of the Renji Hospital, Shanghai Jiao Tong University School of Medicine (RFA2025‐532). Female nude mice with 4–6 weeks were fed for one week to adapt to the environment. KYSE30 cells were injected subcutaneously into the right thigh of the mouse at 3 × 10^6^ cells. When the tumors reached an average volume of 100 mm^3^, the mice were randomly divided into four groups: Ctrl, IR, inhibitor group, or IR combined with inhibitor. Then IR groups were sent to irradiation with 10 Gy X‐rays targeted to the tumor region. From the second day after irradiation, inhibitor of IACS‐010759 (7.5 mg kg^−1^) or dacomitinib (7 mg kg^−1^) was respectively given every other day through orally gavage. Tumor volume was measured three times a week and calculated with the formula of (length × width^^2^)/2.

To establish spontaneous lung metastasis models, 5–6 weeks nude male mice were administered 1.6 × 10⁶ KYSE30 cells with knocked‐down AREG/EREG through tail vein injection. Two months post‐injection, the mice were euthanized, and tumor nodules were examined. For paraffin embedding, the lungs were surgically removed and fixed in formalin overnight. The paraffin‐embedded lung tissues were serially sectioned, subjected to hematoxylin and eosin (H&E) staining, and imaged.

### Chromatin Immunoprecipitation PCR (ChIP‐PCR)

ChIP‐PCR assays were performed using the ChIP Assay Kit (Beyotime, #P2078, China) according to the manufacturer's instructions. Briefly, cells were crosslinked with 1% formaldehyde at 37 °C for 10 min, and added glycine solution for 5 min at room temperature. Then cells were harvested and sonicated. Chromatin was immunoprecipitated by Anti‐CEBPB antibody. IgG is used as a negative control. Primers sequences of ChIP‐PCR and CEBPB binding sites sequence are listed in Table S7 (Supporting Information).

### Luciferase Reporter Assay

Promoters of AREG or EREG were amplified and cloned into pGL3‐Basic vector. The vectors (internal control Renilla LUC, pGL3‐promoter vector, and CEBPB) were transfected into KYSE30 cells and cultured 48 h. The Luciferase activity was measured using a Dual‐Luciferase Reporter Assay System (Promega, E1910) with a Gen5 microplate reader (BIOTEK, USA).

### Statistical Analyses

All data were presented as mean ± SEM or median with lower (Q1‐1.5 × IQR) and upper (Q3 + 1.5 × IQR) whiskers. Student's *t*‐test was applied for the comparison between two groups, and two‐way ANOVA was used for tumor growth and cell growth curves by GraphPad Prism 5.0 Software. *p*‐value was considered statistically significant.

### Ethics Approval and Consent to Participate

This study was approved by the Ethics Committee at Renji Hospital, Shanghai Jiao Tong University School of Medicine (Shanghai, China). Informed consents were obtained from all participants. Animal experiments were approved by the Animal Ethics Committee at Shanghai Cancer Institute, Renji Hospital, Shanghai Jiao Tong University School of Medicine.

## Conflict of Interest

The authors declare no conflict of interest.

## Authors' Contribution

Z.L., M.Y., X.X., and D.Z. contributed equally to this work. Y.H. and X.M. conceived the idea. Y.H., X.M, and G.C. supervised the project. Z.L., M.Y., X.X., and D.Z., performed experiments and analyzed the data. L.X., L.R., X.W., Q. Y., and Y. Z. contributed to clinical sample collection and irradiation of cells or xenograft tumors. Y.D., C.C., J.G., D.Z., and Q. L. contributed to animal experiments. Y.H., G.C., Z.L., and M.Y. wrote and revised the manuscript.

## Supporting information



Supporting Information

## Data Availability

The TCGA data and GEO data referenced during the study are available in a public repository from the TCGA website and NCBI. The RNA‐seq data for KYSE410 and KYSE410R before and after irradiation were deposited in GEO database (GSE293873). All data supporting the findings of this study are available from the corresponding author upon reasonable request.
