# Supplementary material for: Amphiregulin and Epiregulin Confer Radioresistance in Esophageal Squamous Cell Carcinoma Through Oxidative Phosphorylation
Source: Adv Sci (Weinh). 2025 Nov 9;13(6):e07524. doi: 10.1002/advs.202507524 (PMC12866852; doi:10.1002/advs.202507524)
Supplement: Supplementary file 1 — Supporting Information [file ADVS-13-e07524-s001.docx]

Supporting Information

**Amphiregulin and Epiregulin confer** **radioresistance in esophageal squamous cell carcinoma through oxidative phosphorylation**

*Zhang Lin^#^*, *Meilian Yao^#^*, *Xin Xu^#^*, *Dong Zhang^#^*, *Lei Xu*, *Ling Rong*, *Xiaohang Wang*, *Yi Duan*, *Chengkun Chen*, *Jun Gu*, *Yao Zhang, Qiang Liu, Qing Ye, Gaoping Cui*^*^, *Yujun Hao*^*^, *Xiumei Ma*^*^

Z. Lin, M. Yao, D. Zhang, Y. Duan, C. Chen, J. Gu, G. Cui, Y. Hao

State Key Laboratory of Systems Medicine for Cancer, Shanghai Cancer Institute, Renji Hospital, School of Medicine, Shanghai Jiao Tong University, Shanghai, 200032, China

E-mail: cuigaoping@renji.com; yjhao@shsci.org (Yujun Hao)

X. Xu, L. Xu, L. Rong, X. Wang, X. Ma

Department of Radiation Oncology, Renji Hospital, School of Medicine, Shanghai Jiao Tong University, Shanghai, 200127, China

E-mail: maxiumei@renji.com

Y. Zhang

Department of Gastroenterology, Renji Hospital, School of Medicine, Shanghai Jiao Tong University, Shanghai, 200127, China

Q. Liu

Department of Pathology, Renji Hospital, School of Medicine, Shanghai Jiao Tong University, Shanghai, 200127, China

Q. Ye

Department of Thoracic surgery, Renji Hospital, School of Medicine, Shanghai Jiao Tong University, Shanghai, 200127, China

Zhang Lin*^#^*, Meilian Yao*^#^*, Xin Xu*^#^*, and Dong Zhang*^#^* contributed equally to this work.

**
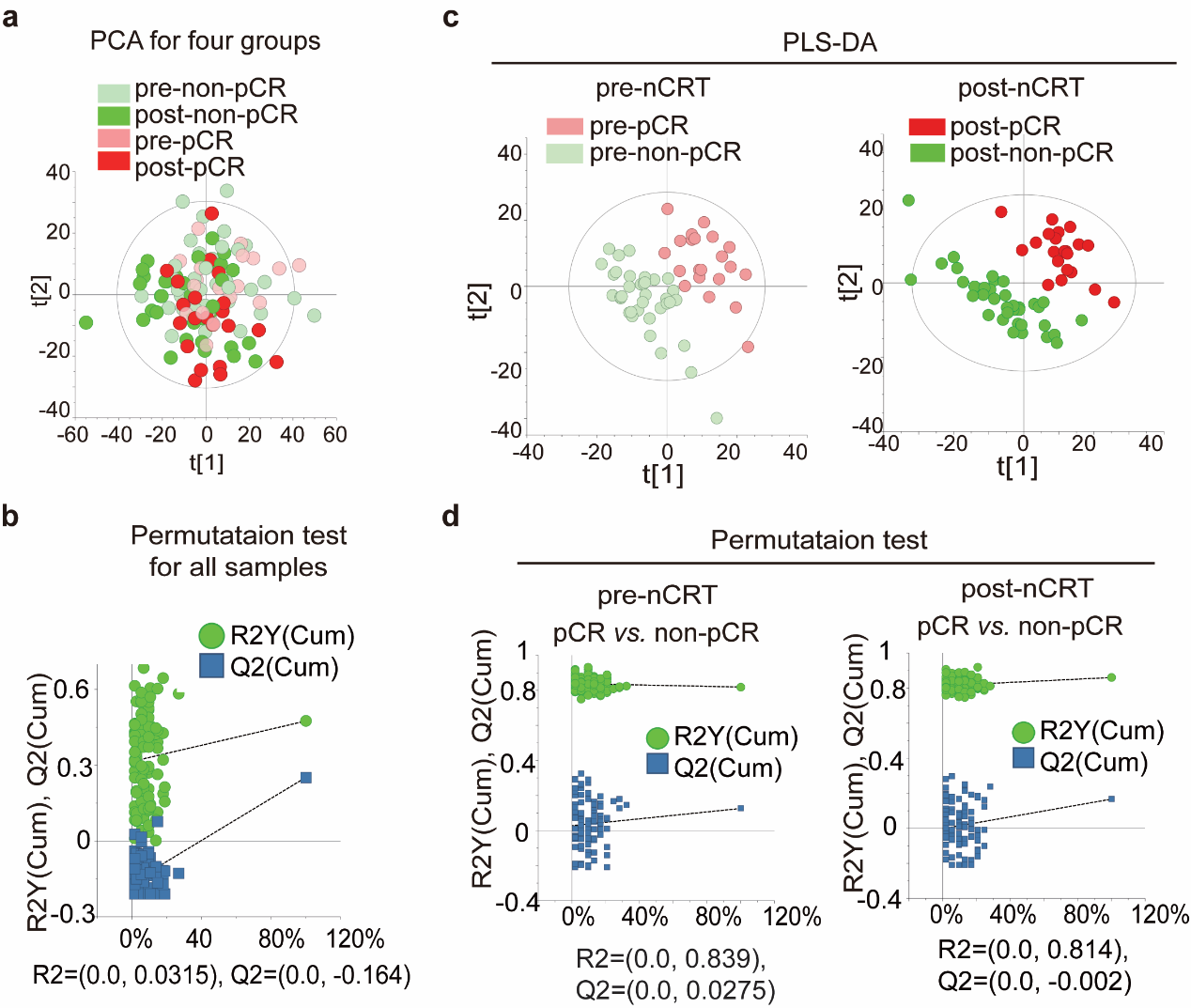
Figure S1 TCA cycle is disturbed in nCRT-responsive ESCC patients.** **a** PCA scores for metabolic profiling of serum samples from nCRT pCR and non-pCR ESCC patients before (pre) or after (post) nCRT (pCR, *n*=23; non-pCR, *n*=36). **b** Permutation tests of PLS-DA models in Figure 1a. **c** PLS-DA models for metabolic profiles from serum samples of ESCC patients before nCRT (pre-pCR vs pre-non-pCR) or after nCRT (post-pCR vs post-non-pCR) (pCR, *n*=23; non-pCR, *n*=36). **d** Permutation tests of PLS-DA models in Figure S1c.

**
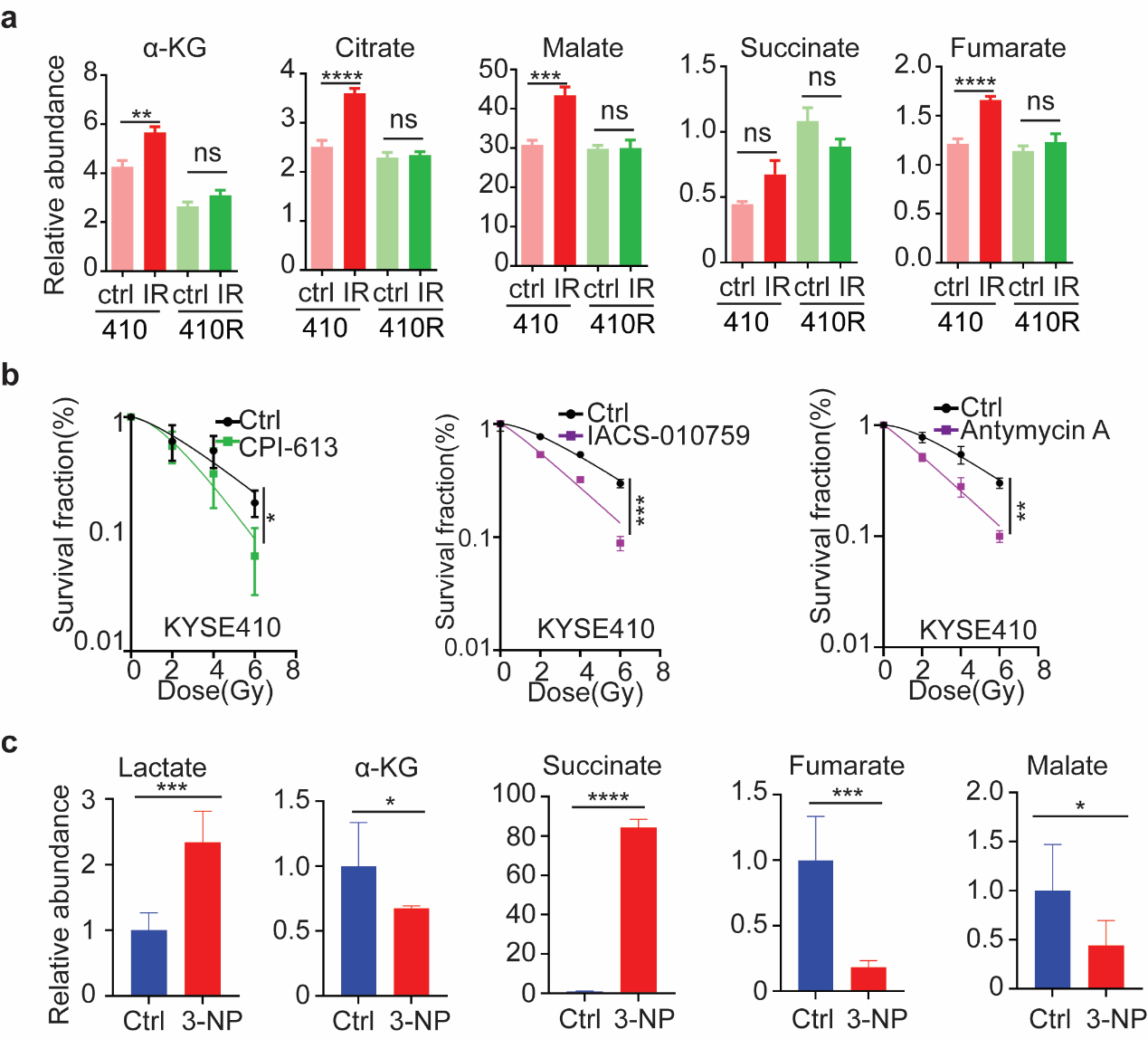
Figure S2** **Inhibition of OXPHOS enhances radiosensitivity of ESCC cells. a** Relative abundance of TCA cycle intermediates in KYSE410 (410) and radioresistant KYSE410R (410R) cells with or without irradiation (IR) were assayed by UHPLC-QTRAP MS (*n*=6). **b** Colony formation survival fractions of KYSE410 cells treated with irradiation (IR) with or without TCA cycle inhibitor (CPI-613) or OXPHOS inhibitors (IACS-010759, Antimycin A) treatment. **c** Relative abundance of TCA cycle intermediates in KYSE30 control cells and 3-NP treatment cells were assayed by UHPLC-QTRAP MS (*n*=6).

Two-way ANOVA was used for statistical analyses of a. Student's t-test was used for statistical analyses of b and c. Data are presented as mean± SEM. * p < 0.05; ** p < 0.01; *** p < 0.001; **** p < 0.0001; ns, not significant.


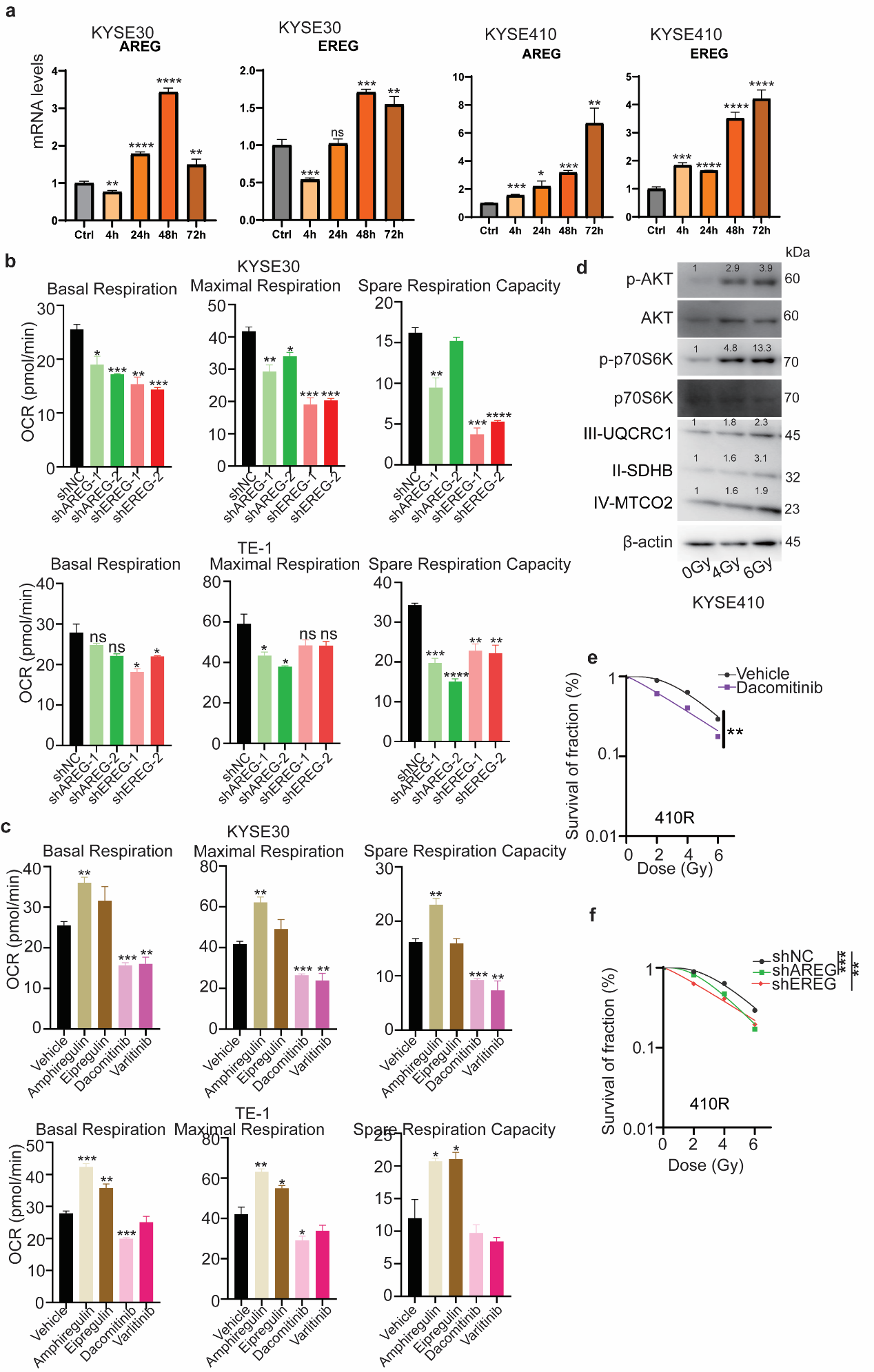
**Figure S3** **AREG and EREG regulate OXPHOS through ErbB signaling pathway. a** Irradiation induced AREG and EREG expression in ESCC cells. **b** Basal and maximal oxygen consumption rate (OCR) of control and AREG or EREG knockdown cells were measured by Seahorse analyzer. **c** Basal and maximal oxygen consumption rate (OCR) of control and AREG (Amphiregulin), EREG (Eipregulin), Dacomitinib or Varlitinib treated cells were measured by Seahorse analyzer. **d** AKT/mTOR/mitochondrial complex proteins axis was responded to irradiation in dose dependent manner. **e** ErbB inhibitors (Dacomitinib) treatment increased radiosensitivity in 410 IR-tolerant cells (410R). **f** Knockdown of AREG or EREG increased radiosensitivity of 410 IR-tolerant cells (410R).

Student's t-test was used for statistical analyses of a and b. Data are presented as mean± SEM. * p < 0.05; ** p < 0.01; *** p < 0.001; **** p < 0.0001; ns, not significant.


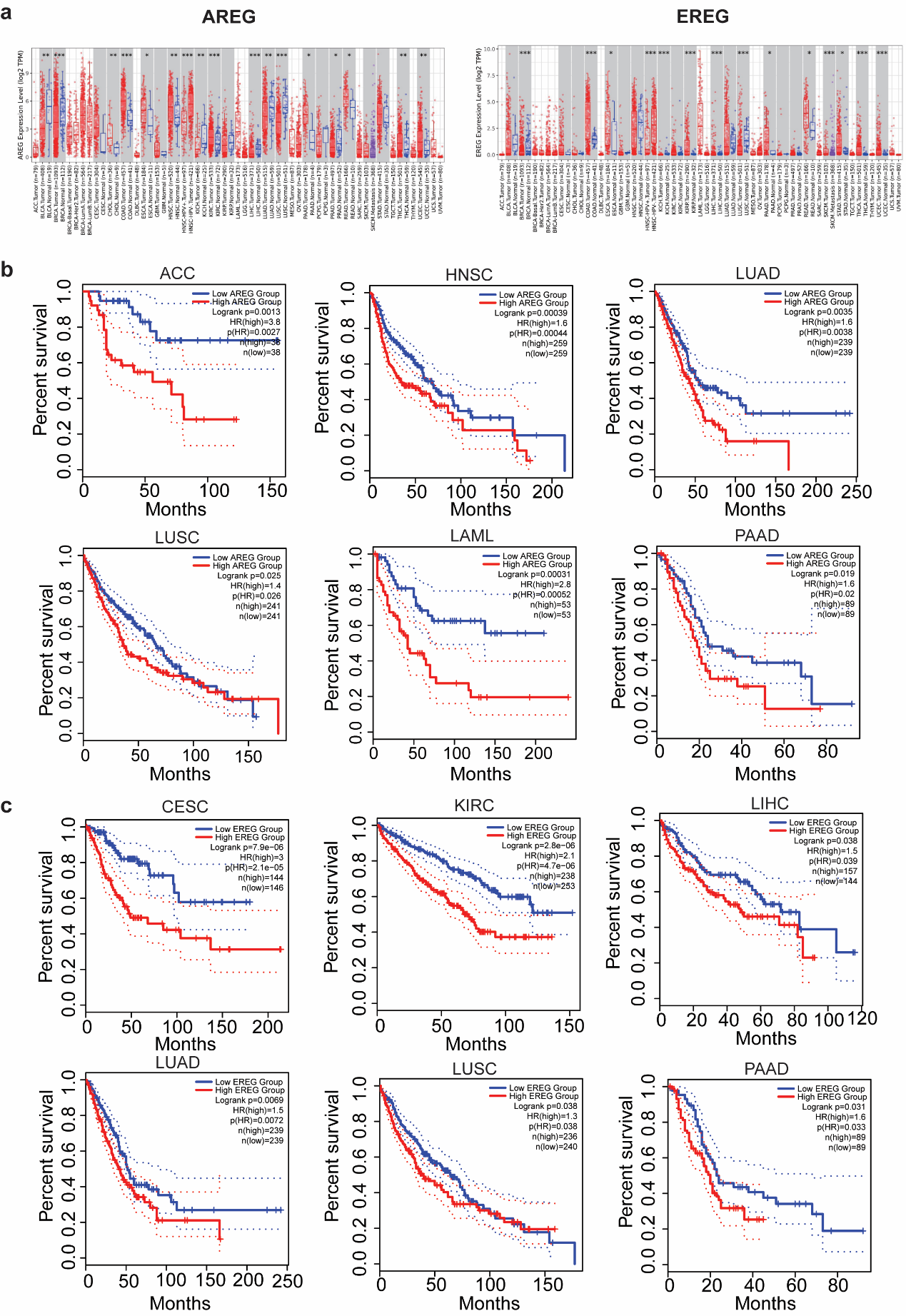
**Figure S4** **AREG and EREG are oncogenes. a** AREG and EREG were highly expressed in tumor samples compared with normal tissue samples across malignances (TIMER2.0). **b** High levels of AREG were correlated with worse overall survival of cancer patients (GEPIA). **c** High levels of EREG were correlated with worse overall survival of cancer patients (GEPIA).

Student's t-test was used for statistical analyses of a. * p < 0.05; ** p < 0.01; *** p < 0.001; **** p < 0.0001.


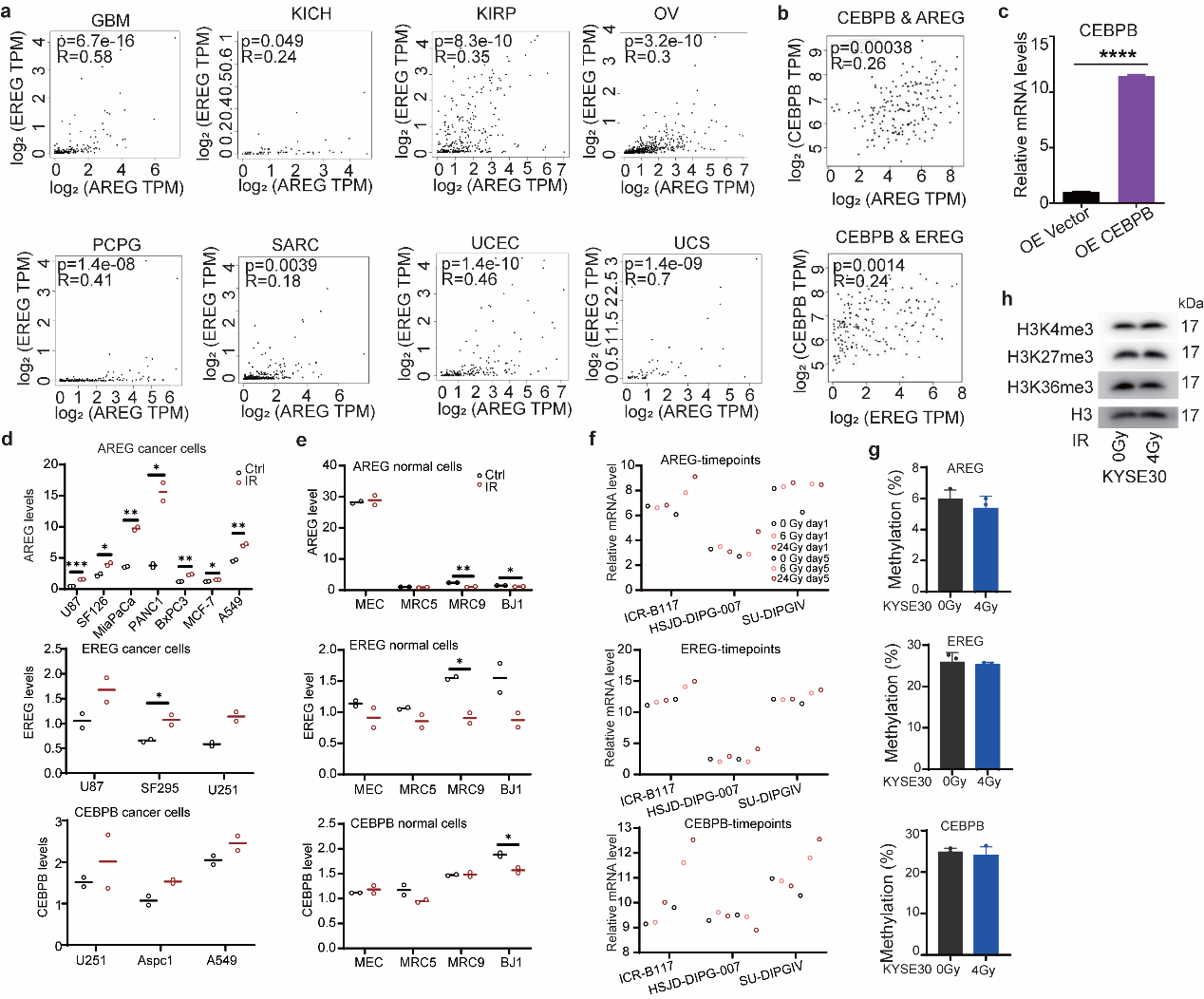
**Figure S5 AREG and EREG are co-expressed across malignances. a** The expression of AREG and EREG was highly correlated across malignances in TCGA database. **b** The expression of CEBPB was positively correlated with the expression of AREG or EREG in TCGA ESCA dataset. **c** qRT-PCR analysis showed the overexpression efficiency of CEBPB. **d** The expression of AREG, EREG, or CEBPB in control cancer cells (Ctrl) and irradiation-treated cancer cells (IR) were analyzed according to GSE10547. **e** The expression of AREG, EREG, or CEBPB in control normal cells (Ctrl) and irradiation-treated normal cells (IR) were analyzed according to GSE10547. **f** Irradiation induces the expression of AREG, EREG, and CEBPB in a time-dependent manner according to GSE291046 dataset. **g** Irradiation had no impact on DNA methylation levels on promoters of AREG, EREG, or CEBPB. **h** Irradiation had no impact on several tri-methylation of histone H3 in ESCC cells.

Student's t-test was used for statistical analyses of c, d, e. Data are presented as mean± SEM. * p < 0.05; ** p < 0.01; *** p < 0.001; **** p < 0.0001.


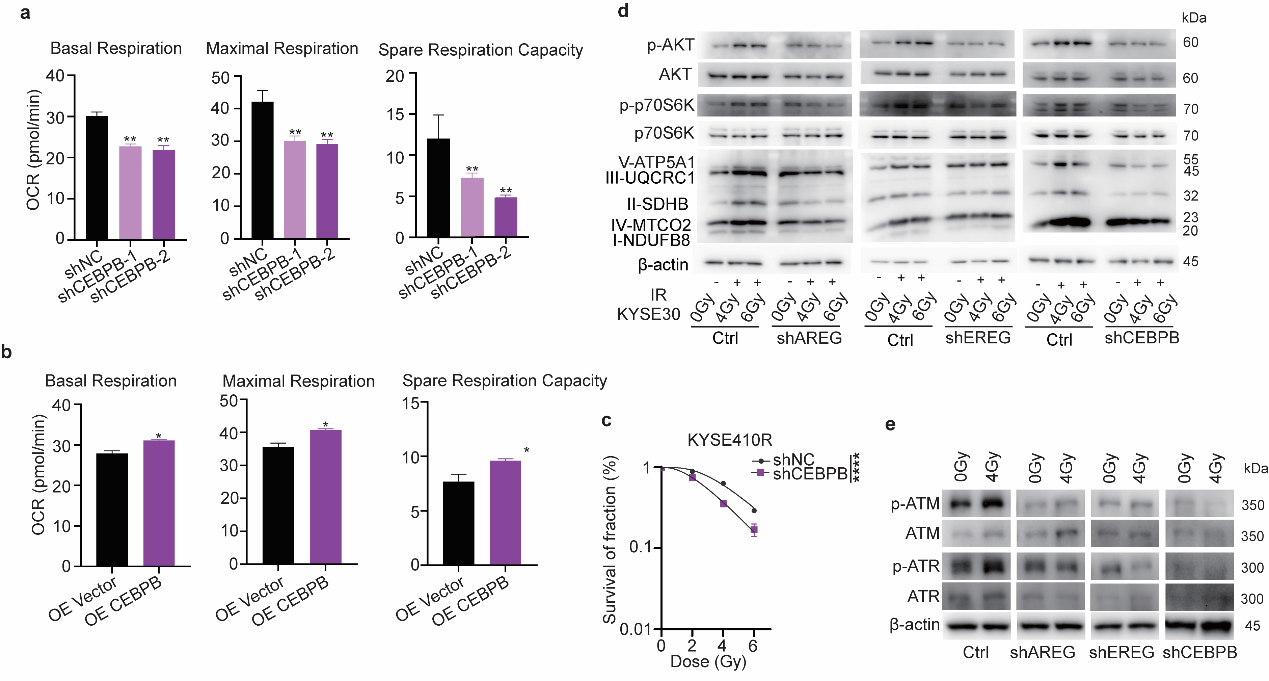
**Figure S6** **CEBPB regulates OXPHOS. a** Basal and maximal oxygen consumption rate (OCR) of control and CEBPB knockdown cells were measured by Seahorse analyzer. **b** Basal and maximal oxygen consumption rate (OCR) of control and CEBPB overexpression cells were measured by Seahorse analyzer. **c** Knockdown of CEBPB increased radiosensitivity of 410 IR-tolerant cells (410R). **d** The induction of AKT/mTOR/mitochondrial complex axis by irradiation were blocked by knockdown of AREG, EREG or CEBPB. **e** The induction of DNA damage repair pathway (pATM and pATR) by irradiation were blocked by knockdown of AREG, EREG or CEBPB.

Student's t-test was used for statistical analyses. Data are presented as mean± SEM. * p < 0.05; ** p < 0.01; **** p < 0.0001.


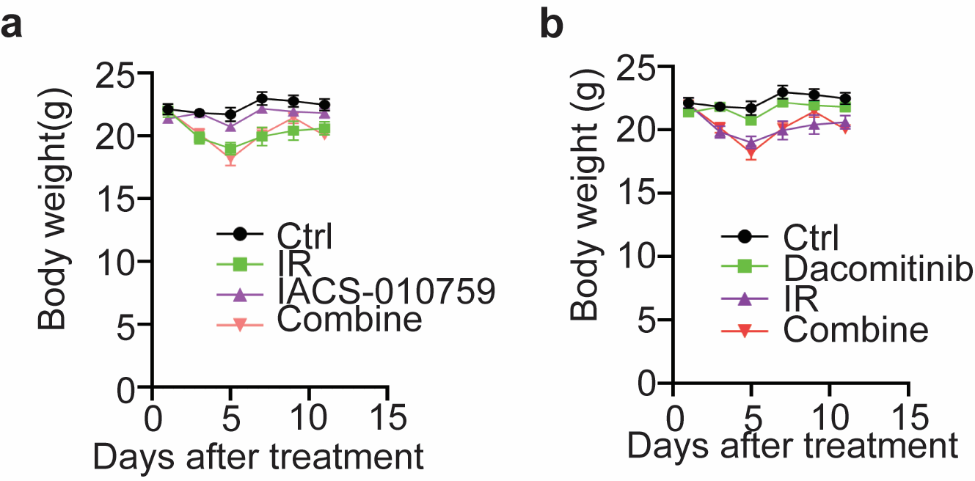
**Figure S7** **Combination of irradiation with IACS-010759 (a) or** **dacomitinib (b)** **have no effect on mice body weights.**

**Table S1 Comparison of clinical features at baseline for pCR and non-pCR ESCC patients.**

| **Clinical**  **characteristics** | **Histological response** | | ***p* value** |
| --- | --- | --- | --- |
|  | pCR [*n*/%]  N=23 | non-pCR [*n*/%]  N=36 |  |
| Age |  |  | 0.43 |
| >=65 | 11 (47.8) | 16 (44.4) |  |
| <65 | 12 (52.2) | 20 (55.6) |  |
| Gender |  |  | 0.13 |
| Male | 17 (73.9) | 33 (91.7) |  |
| Female | 6 (26.1) | 3 (8.3) |  |
| Tumor location |  |  | 0.84 |
| Upper thoracic | 2 (8.7) | 3 (8.3) |  |
| Middle thoracic | 8 (34.8) | 10 (27.7) |  |
| Lower thoracic | 13 (56.5) | 23 (63.8) |  |
| Length |  |  | 0.78 |
| <5cm | 6 (26.1) | 11 (30.6) |  |
| >=5cm | 17 (73.9) | 25 (69.4) |  |
| T stages |  |  | 0.93 |
| T2 | 1 (4.3) | 1 (2.8) |  |
| T3 | 21 (91.3) | 33 (91.7) |  |
| T4 | 1 (4.3) | 2 (5.6) |  |
| N stages |  |  | 0.86 |
| N0 | 2 (8.7) | 1 (2.8) |  |
| N1 | 14 (60.9) | 14 (38.9) |  |
| N2 | 7 (30.4) | 20 (55.6) |  |
| N3 | 0 (0) | 1 (2.8) |  |
| TNM stages |  |  | 0.28 |
| Ⅱ | 3 (28.2) | 1 (2.8) |  |
| Ⅲ | 19 (68.2) | 32 (88.9) |  |
| Ⅳa | 1 (3.6) | 3 (8.3) |  |

Chi-square test was used for statistical analyses.

**Table S2 The DEMs identified by untargeted metabolomics**

| **Untargeted detection** | | **DEMs** |
| --- | --- | --- |
| pCR *vs.* non-pCR  (for pre-nCRT) | 3β-Hydroxy-5-cholenoic acid, N-α-acetyllysine, Taurine, Erucic acid | |
| pCR *vs.* non-pCR  (for post-nCRT) | 7-Methylxanthine, 3-Phenylbutyric acid, D-α-aminobutyric acid, L-Asparagine, L-Glutamic acid | |
| pre-nCRT *vs.* post-nCRT (for pCR) | Citrulline, Homo-L-arginine, Taurine, Ornithine, Inosine, Leucinic acid, Mandelic acid, Dimethylmalonic acid, Quinic acid, 4-Ethylbenzoic acid | |
| pre-nCRT *vs.* post-nCRT (for non-pCR) | Citrulline, D-α-aminobutyric acid, L-Alanine, Taurine, Ornithine, Inosine, Leucinic acid, Uridine, L-Histidine, L-Asparagine, Homo-L-arginine, 4-Hydroxyproline, Quinic acid, Taurodeoxycholic acid, Guanosine, Uridine 5'-monophosphate,  Erucic acid, 4-Ethylbenzoic acid, D-Arginine, Glucaric acid, Pyroglutamic acid, Ascorbic acid, L-Threonine | |

| **Targeted detection** | **DEMs** |
| --- | --- |
| pCR *vs.* non-pCR (for pre-nCRT) | F6P, Uric acid, CDP |
| pCR *vs.* non-pCR (for post-nCRT) | N-acetyl-L-ornithine, AICAR, Adenosine, Ornithine, Malate, 2-Hydroxyglutarate, Dihydroxyacetone-phosphate, Pyruvate, sn-glycerol-3-phosphate, α-ketoglutarate, FMN, Uridine, Fumarate, 6-Phospho-D-gluconate, Xanthine, Lactate, Dihydroorotate |
| pre-nCRT *vs.* post-nCRT (for pCR) | Citrulline, Cystine, SAM, Spermidine, L-arginino-succinate, Betaine, Taurine, Adenosine, Pyruvate, D-glyceraldehdye-3-phosphate, Succinate, Malate, Lactate, 2-Deoxyglucose-6-phosphate, F6P, Carbamoyl phosphate, Dihydroxyacetone-phosphate, α-ketoglutarate, FMN, Deoxyribose-phosphate, Uridine, 6-Phospho-D-gluconate, G6P, 2-Hydroxyglutarate, Aconitate, sn-glycerol-3-phosphate, cAMP, Uracil, Uric acid, Fumarate |
| pre-nCRT *vs.* post-nCRT (for non-pCR) | Citrulline, Cystine, SAM, Spermidine, AICAR, Hypoxanthine, Leucine/Isoleucine, Ornithine, Proline, Guanosine |

**Table S3 The DEMs identified by targeted metabolomics**

**Table S4 Enriched KEGG pathways for DEMs of pre-pCR *vs*. post-pCR groups**

| **Pathway Name** | **Match Status** | ***p* value** | **Hit metabolites** |
| --- | --- | --- | --- |
| Arginine biosynthesis | 6/14 | 4.89E-8 | N-(L-Arginino) succinate; L-Citrulline; Carbamoyl phosphate; L-Ornithine; 2-Oxoglutarate; Fumarate |
| TCA cycle | 6/20 | 5.86E-7 | 2-Oxoglutarate; Succinate; Malate; cis-Aconitate; Pyruvate; Fumarate |
| Alanine, aspartate and glutamate metabolism | 6/28 | 5.16E-6 | Fumarate; Pyruvate; Succinate; 2-Oxoglutarate; Carbamoyl phosphate; N-(L-Arginino) succinate |
| Glycolysis/  Gluconeogenesis | 5/26 | 6.22E-5 | Pyruvate; Lactate; D-Fructose 6-phosphate; D-Glyceraldehyde 3-phosphate; Dihydroxyacetone phosphate |
| Pyruvate metabolism | 4/23 | 5.61E-4 | Pyruvate; Malate; Lactate; Fumarate |
| Pentose phosphate pathway | 4/23 | 5.40E-4 | 2-Deoxy-D-ribose 5-phosphate; D-Glyceraldehyde 3-phosphate; D-Fructose 6-phosphate; 6-Phospho-D-gluconate |
| Arginine and proline metabolism | 4/36 | 3.06E-03 | L-Ornithine; Spermidine; S-Adenosyl-L-methionine; Pyruvate |

Hypergeometric Test was used for statistical analyses.

**Table S5 Enriched KEGG pathways for DEMs of pre-non-pCR *vs*. post-non-pCR groups**

| **Pathway Name** | **Match Status** | ***p* value** | **Hit metabolites** |
| --- | --- | --- | --- |
| Arginine and proline metabolism | 5/36 | 6.97E-5 | Spermidine; Hydroxyproline; L-Proline; L-Ornithine S-Adenosyl-L-methionine |
| Valine, leucine and isoleucine biosynthesis | 2/8 | 4.43E-3 | L-Threonine; L-Leucine |
| Glutathione metabolism | 3/28 | 5.25E-3 | Pyroglutamic acid; L-Ornithine; Spermidine |
| Purine metabolism | 4/70 | 1.16E-2 | AICAR; Hypoxanthine; Inosine; Guanosine |
| Arginine biosynthesis | 2/14 | 1.37E-2 | L-Citrulline; L-Ornithine |

Hypergeometric Test was used for statistical analyses.

**Table S6 The list of antibodies used in this study**

| **Antibodies** | **Company** | **Catalog No.** |
| --- | --- | --- |
| Rabbit polyclonal antibody anti-Akt | Cell Signaling Technology | Cat# 9272, RRID: AB_329827 |
| Rabbit monoclonal antibody anti-p-Akt kinase (473) | Cell Signaling Technology | Cat# 4060, RRID: AB_2716452 |
| Rabbit polyclonal antibody anti-p-p70S6 kinase (Ser371) | Cell Signaling Technology | Cat# 9208, RRID: AB_330990 |
| Rabbit monoclonal antibody anti-p70S6 kinase | Cell Signaling Technology | Cat# 2708, RRID: AB_390722 |
| Rabbit polyclonal antibody anti-Actin | Sigma-Aldrich | Cat# A2066, RRID: AB_476693 |
| Rabbit polyclonal antibody anti-ATP5A1 | Proteintech | Cat# 14676-1-AP |
| Rabbit polyclonal antibody anti-UQCRC1 | Proteintech | Cat# 21705-1-AP |
| Rabbit polyclonal antibody anti-SDHB | Proteintech | Cat# 10620-1-AP |
| Rabbit polyclonal antibody anti-MTCO2 | Proteintech | Cat# 55070-1-AP |
| Mouse antibody anti-Phospho-Histone H2A.X (Ser139) (D7T2V) | Cell Signaling Technology | Cat# 80312 RRID: AB_2799949 |
| Rabbit Monoclonal Antibody anti-ATM | Beyotime | Cat# AF1399 |
| Rabbit polyclonal antibody anti-Phospho-ATM (Ser1981) | Beyotime | Cat# AF5743 |
| Rabbit Polyclonal Antibody anti-ATR | Beyotime | Cat# AF6267 |

**Table S7 Primers and oligos used in this study**

| **Primer name** | **Primer sequences（5’ to 3’）** | |
| --- | --- | --- |
| **Primers for** **qRT-PCR** | | |
| AREG | Forward  Reverse | GTGGTGCTGTCGCTCTTGATA  CCCCAGAAAATGGTTCACGCT |
| EREG | Forward  Reverse | GGACAGTGCATCTATCTGGTGG  TTGGTGGACGGTTAAAAAGAAGT |
| CEBPB | Forward  Reverse | AGCGACGAGTACAAGATCCG  GCTTGAACAAGTTCCGCAGG |
| *β*-actin | Forward | CATGTACGTTGCTATCCAGGC |
|  | Reverse | CTCCTTAATGTCACGCACGAT |
| **Primers for ChIP-qPCR** |  |  |
| AREG-promoter-motif-1 | Forward | GGGGATTTGAAATTCTGGCCAC |
|  | Reverse | AGTACCTGACAGAAGTCCAG |
| AREG-promoter-motif-2 | Forward | AGACCAACTGCGTGAGCTCCAA |
|  | Reverse | CTGTGGAAAGATGAAGAGGC |
| AREG-promoter-motif-3 | Forward | CTATCACCCATGATGGAGTAC |
|  | Reverse | AGTGGCTCATGCCTGTAATC |
| EREG-promoter-motif-1 | Forward | AAGCCATCTGGTCCACGCT |
|  | Reverse | GAGCTCAACTGTCTGGTGCT |
| EREG-promoter-motif-2 | Forward | GTACCACCATCTTAAGC |
|  | Reverse | GAGCTTGCAGTGAGTCGAGAT |
| EREG-promoter-motif-3 | Forward | ACGACTCCATCAGCATAGGCA |
|  | Reverse | CTTCCAATGTGGCTCAGGCAT |
| **CEBPB predict binding site for ChIP-qPCR** | | |
| AREG-motif-1 | AATTGCTTCAT(-429~-419) | |
| AREG-motif-2 | AATTGCCTCAT(-1915~-1905) | |
| AREG-motif-3 | ATGGCAAAAC(-1175~-1166) | |
| EREG-motif-1 | AGTTTCATAAT(-91~-81) | |
| EREG-motif-2 | AATTTCCTCAT(-1746~-1736) | |
| EREG-motif-3 | TATTTCCCCAG(-779~-769) | |
| **Subcloning primers** |  |  |
| pGL3 AREG promoter | Forward | GCCAGAACATTTCTCTATCGATAGGTACCAGACCAACTGCGTGAGCTCCAA |
|  | Reverse | CCAACAGTACCGGAATGCCAAGCTTGTAGGGCGGCGCGCACCTGC |
| pGL3 EREG promoter | Forward | GCCAGAACATTTCTCTATCGATAGGTACCAGTCACTCAATGCCTGCTTTC |
|  | Reverse | CCAACAGTACCGGAATGCCAAGCTTGAGCTCAACTGTCTGGTGCT |
| **Sequence for shRNAs** | | |
| AREG shRNA-1 | Forward  Reverse | GAACGAAAGAAACTTCGACAA  TTGTCGAAGTTTCTTTCGTTC |
| AREG shRNA-2 | Forward  Reverse | GAACCACAAATACCTGGCTAT  ATAGCCAGGTATTTGTGGTTC |
| EREG shRNA-1 | Forward | GCCACATAATAACCCAGTTAA |
|  | Reverse | TTAACTGGGTTATTATGTGGC |
| EREG shRNA-2 | Forward | CGTGTGGCTCAAGTGTCAATA |
|  | Reverse | TATTGACACTTGAGCCACACG |
| CEBPB shRNA-1 | Forward  Reverse | CCCGTGGTGTTATTTAAAGAA  TTCTTTAAATAACACCACGGG |
| CEBPB shRNA-2 | Forward  Reverse | CGACTTCCTCTCCGACCTCTT  AAGAGGTCGGAGAGGAAGTCG |
| shRNA-NC | Forward  Reverse | GCTTCGCGCCGTAGTCTTA  TAAGACTACGGCGCGAAGC |
